# Efficacy of Huangqi Injection in the Treatment of Hypertensive Nephropathy: A Systematic Review and Meta-Analysis

**DOI:** 10.3389/fmed.2022.838256

**Published:** 2022-04-25

**Authors:** ZhongChi Xu, LiChao Qian, RuGe Niu, Ying Yang, ChunLing Liu, Xin Lin

**Affiliations:** ^1^Jiangsu Province Hospital of Chinese Medicine, Affiliated Hospital of Nanjing University of Chinese Medicine, Nanjing, China; ^2^Nanjing Hospital of Chinese Medicine, Affiliated Hospital of Nanjing University of Chinese Medicine, Nanjing, China

**Keywords:** hypertensive nephropathy, Astragalus membranaceus (Fisch.) Bunge, Huangqi injection, systematic review, meta-analysis

## Abstract

**Background:**

Huangqi injection (HQI) is the extract of Astragalus membranaceus (Fisch.) Bunge, which is widely used in the treatment of a variety of diseases in China. It is supposed to be an important adjuvant therapy for hypertensive nephropathy.

**Objective:**

To evaluate the efficacy of HQI combined with antihypertensive drugs in the treatment of hypertensive nephropathy.

**Materials and Methods:**

We systematically searched China National Knowledge Infrastructure (CNKI), Chinese Scientific Journals Database (VIP), Wanfang Knowledge Service Platform (WanfangData), Chinese Biomedical Database (CBM), EMBASE, PubMed and Cochrane Library from their inception to April 23st, 2021. All studies were independently screened by two auditors according to the inclusion and exclusion criteria. Randomized controlled trials comparing HQI in combination with antihypertensive drugs vs. antihypertensive drugs alone were extracted.

**Results:**

The meta-analysis included 15 studies involving 1,483 participants.The effect of HQI combined with antihypertensive drugs is better than that of antihypertensive drugs alone in regulating hypertensive nephropathy for reducing 24-h urinary total protein (24 h UTP) [WMD=-0.29, 95% CI (−0.40, −0.18), *P* = 0.000], microalbuminuria (mALB) [WMD = −17.04, 95% CI (−23.14, −10.94), *P* = 0.000], serum creatinine (SCr) [WMD = −40.39, 95% CI (−70.39, −10.39), *P* = 0.008], systolic blood pressure (SBP) [WMD = −9.50, 95% CI (−14.64, −4.37), *P* = 0.000], diastolic blood pressure (DBP) [WMD = −4.588, 95% CI (−6.036, −3.140), *P* = 0.000], cystatin-C (Cys-c) [WMD = −0.854, 95% CI (−0.99, −0.72), *P* = 0.000], blood urea nitrogen (BUN) [WMD = −4.155, 95% CI (−6.152, −2.157), *P* = 0.000].

**Conclusion:**

The combination of HQI and antihypertensive drugs is more efficient in improving the related indexes of patients with hypertensive nephropathy than using antihypertensive drugs alone, and a moderate dose of HQI (no more than 30 mL) may benefit more. However, the quality of the methodology is low and the number of samples is small, the results need to be confirmed by more stringent randomized controlled trials.

## Introduction

Hypertension is one of the important risk factors for cardiovascular events and kidney disease, which is considered to be a potential cause of death and a major health problem in all regions of the world. The prevalence of hypertension in different countries ranges from 22 to 55%, which is expected to increase to 60% by 2025. It has become a global high-burden disease (1). Chronic kidney disease (CKD) is a common complication in patients with hypertension, which plays a major role in the progression of most forms of CKD, including diabetic nephropathy. Hypertension accelerates the progression of kidney disease, and the deterioration of renal function makes it more difficult to control blood pressure, resulting in a vicious circle of progressive renal failure (2, 3). When the glomerular filtration rate (GFR) falls below the critical level, CKD will continue to develop to end-stage renal disease (ESRD). Hypertensive nephropathy is the main cause of ESRD after diabetic nephropathy (4). In the United States, hypertension is the second major cause of ESRD patients. More than 30,000 people are diagnosed with hypertension-related ESRD every year, and the number of patients diagnosed with RSRD continues to grow steadily, which has become a major challenge in the field of public health care (5–7).

Studies found that hypertension is positively related to the occurrence and development of cardiovascular disease and kidney disease. Considering that reducing blood pressure can significantly reduce the risk of chronic kidney disease, active intervention and management of blood pressure should be carried out in patients with hypertension. Moreover, the development of hypertensive nephropathy is related to many factors, such as sympathetic nervous activity (SNA) change, renin-angiotensin-aldosterone system (RAAS) activation, arteriosclerosis, water and sodium retention, and genetic susceptibility (3). Current guidelines recommend that adults with hypertension and chronic kidney disease should control the blood pressure below 130/80 mmHg, with an emphasis on the management of blood pressure and urinary microalbumin, and prefer to RAAS inhibitor drugs, usually in combination with diuretics or calcium antagonists to slow the progression of kidney disease (8). Microalbumin (mALB) which was defined as the excretion rate of urinary albumin between 20–200 mg/min or 30–299 mg/d is an important indicator of cardiovascular events and renal function. The degree of mALB is closely related to the progression of ESRD (9, 10). Studies have shown that after active treatment, mALB can be reduced by more than 30%, while the risk of dialysis in 3–5 years is reduced by 39–72%. Progressive kidney disease can be minimized when albuminuria and blood pressure decrease simultaneously (11, 12). As a part of alternative medical adjuvant therapy, traditional chinese medicine is widely used in the treatment of hypertension and chronic kidney disease. More and more evidence support the point of view (13–15).

Huangqi injection (HQI) is a Chinese herbal medicine which is a water extraction and sterilization solution of dried roots of Astragalus membranaceus (Fisch.) Bunge. HQI has a wide range of pharmacological effects. Ultra-high performance liquid phase tandem quadrupole time-of-flight mass spectrometry has identified 46 active components of HQI, such as saponins, flavonoids and amino acids (16, 17). Studies have shown that HQI can dilate blood vessels, increase coronary and renal blood perfusion, reduce myocardial oxygen consumption, improve renal microcirculation, eliminate lipid peroxides and scavenge ROS (18–20). HQI can inhibit phosphodiesterase activity, reduce cAMP decomposition, increase extracellular calcium (CA^2+^) inflow and sarcoplasmic reticulum CA^2+^ outflow, increase cardiomyocyte excitability, thus enhance myocardial contractility. HQI can inhibit thromboxane synthesis, reduce blood viscosity, alleviate water retention and increase eGFR by improving arginine vasopressin (AVP) system and AVP-dependent aquaporin2 levels (21). With the study of the pharmacological effects of Astragalus membranaceus (Fisch.) Bunge, HQI is widely used in clinical practice in China, such as in the treatment of coronary heart disease, cardiomyopathy, acute and chronic glomerulonephritis, diabetic nephropathy, as well as hypertensive nephropathy (20, 22).

In recent years, there have been many clinical practices comparing the efficacy of HQI combined with antihypertensive drugs with that of antihypertensive drugs alone in the treatment of hypertensive nephropathy, and the results show that the combined use of HQI and antihypertensive drugs benefits patients, but the efficacy of HQI in the treatment of hypertensive nephropathy has not been laborated. Therefore, we conducted a systematic review and meta-analysis to determine the efficacy of HQI in adjuvant treatment of hypertensive nephropathy.

## Materials and Methods

Systematic reviews and meta-analyses were designed in accordance with the guidelines of the 2009 Preferred Reporting Project for Systematic Analysis and Meta-analysis (PRISMA) statement (23).

### Search Strategy

Literature retrieval is carried out from the electronic network databases from inception to April 23st, 2021, and the retrieval language is not limited. The databases include China National Knowledge Infrastructure (CNKI), China Scientific Journal Database (VIP), WanfangData Knowledge Service Platform (WanfangData), Chinese Biomedical Database (CBM), EMBASE, Cochrane Library and PubMed. The retrieval scheme was based on the combination of subject words and free words, and the search terms included “hypertension,” “Hypertensive nephropathy,” “Hypertension nephropathy,” “Hypertensive renal injury,” “Hypertensive renal damage,” “Hypertensive kidney injury,” “Hypertensive kidney damage,” “Astragalus,” “Astragalus injection,” “Huangqi” and “Huangqi Injection.”

### Inclusion Criteries

Inclusion studies should meet the following criteria: (1) Randomized Controlled Trials (RCTs) regardless of blinding, publication status, type of publication, or language; (2) Patients meeting the diagnostic criteria of hypertensive nephropathy, ①meeting the diagnostic criteria of hypertension. Hypertension was defined as SBP ≥ 140 mmHg or DBP≥90 mmHg, ②appearing clinical patterns of abnormal renal function, such as increased urinary protein and serum creatinine, ③excluding secondary hypertension and primary renal disease caused by other reasons, other serious diseases or complications; (3) Comparing the intervention with HQI combined with antihypertensive drugs with the treatment in the control group. The intervention measures in the control group included antihypertensive drugs and conventional therapy, and there were no restrictions on the dosage, type, frequency or course of treatment.

### Exclusion Criteria

Research that meets the following criteria will be excluded : (1) duplicate publications; (2) basic research, non-clinical studies, systematic review, case report and case discussion; (3) use of any other drugs and/or herbal medicines during the study; (4) clinical trials from which relevant data cannot be extracted; (5) clinical trials that did not meet the expected inclusion criteria.

### Study Selection

The levels of serum creatinine (Scr), microalbuminuria (mALB) and 24-h urinary total protein (24 h UTP) were selected as the main outcome indicators. The secondary outcome included blood urea nitrogen (BUN), cystatin C (Cys-c), systolic blood pressure (SBP), diastolic blood pressure (DBP).

### Data Extraction

Two researchers independently conducted searches according to the search strategy. Preliminary screening was based on topics and abstracts, excluding studies that were obviously unqualified. For studies that might be eligible, full text screening was performed according to inclusion and exclusion criteria, and data was extracted. Two researchers then cross-checked the studies.Any differences are resolved through discussion or final arbitration verified by a third researcher.

### Quality Assessment

According to the bias risk assessment tool in the Cochrane Handbook for Systematic Reviews, the methodological quality of the included study was evaluated by two researchers. The risk assessment tool consists of seven items: (1) generation of random sequences; (2) allocation concealment; (3) blinding of participants and personnel; (4) blinding of outcome data; (5) incomplete outcome data; (6) selective reporting; (7) other biases, These items were assessed as having “high bias risk,” “low bias risk” or “unclear bias risk” according to the evaluation criteria.

### Data Analysis

Stata14.0 software was used to analyze the meta-analysis. For continuous variables, weighted mean difference (WMD) or standardized mean difference (SMD) and 95% CI were used. Heterogeneity is evaluated using *I*^2^ statistics and χ^2^ statistics. The effect model was selected according to the results of heterogeneity test, and the fixed effect model was used when *P* ≥ 0.05 and *I*^2^ <50. *P* < 0.05 and *I*^2^≥ 50 indicated that the heterogeneity of the results could not be ignored, and we used the random effect model. *P* < 0.05 was considered to be statistically significant. Potential publication bias was tested by egger. The possible abnormal studies were evaluated by sensitivity analysis, and the results were compared with the meta-analysis before exclusion to determine how the excluded study would affect the size of the merger effect and the stability of the meta-analysis. Then we analyzed or eliminated the possible sources of heterogeneity. The indicators with high heterogeneity could not be excluded by subgroup analysis in order to explore the potential causes of heterogeneity according to different interventions or other factors.

## Results

### Search Results

**Figure 1:**
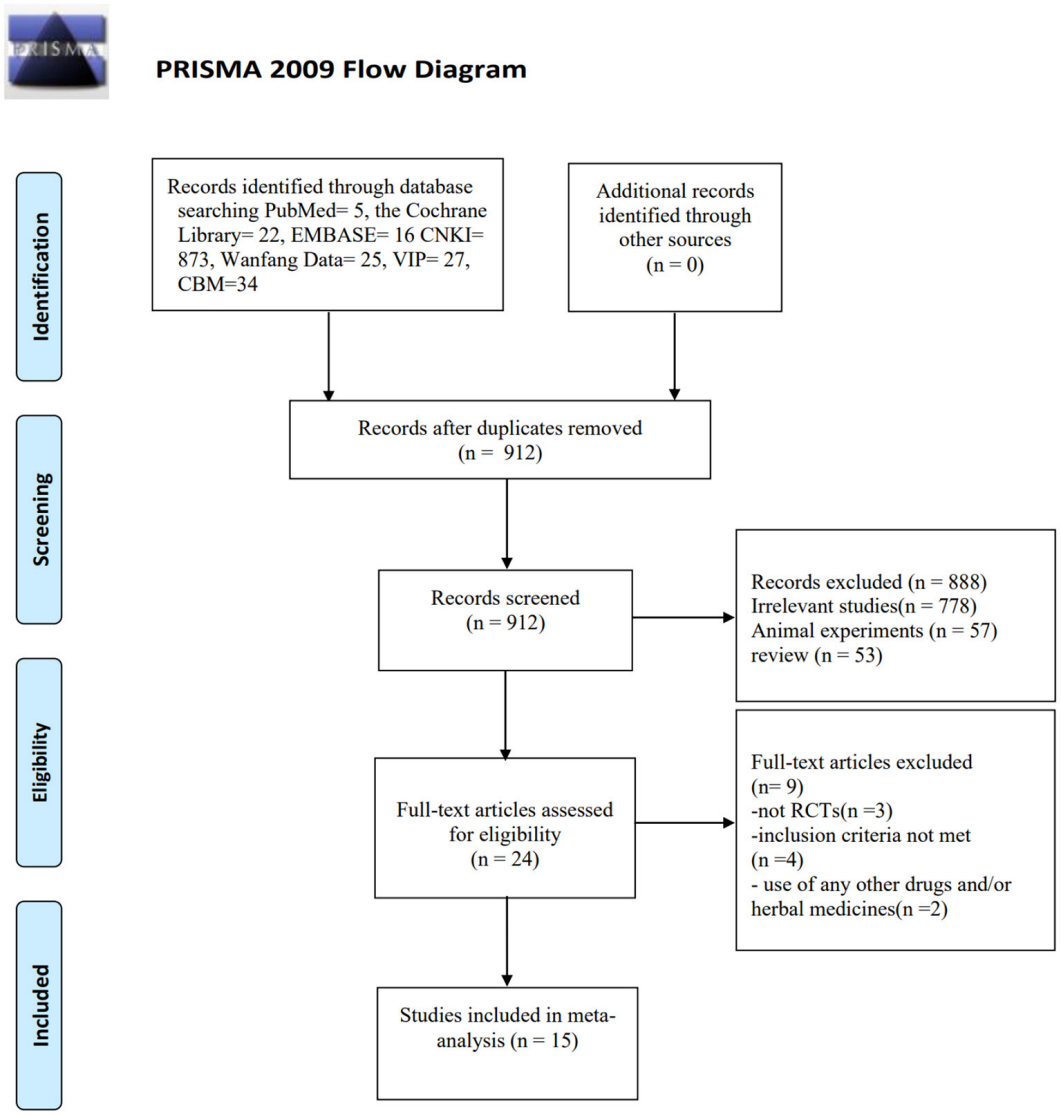
Flowchart of the process for literature retrieval.

A total of 1002 articles [Cochrane Library (*n* = 22), PubMed (*n* = 5), EMBASE (*n* = 16), CBM (*n* = 34), CNKI (*n* = 873), WanfangData (*n* = 25) and VIP (*n* = 27)] met the criteria through the search strategy, and 90 of them were excluded due to repeated publication. Eight hundred and eighty-eight articles were excluded after reviewing titles and abstracts. The remaining 24 articles were reviewed in full, a further 9 articles were excluded. Among them, 3 were not RCTs, 4 were not in accordance with the inclusion criteria, and 2 used any other drugs and/or herbal medicines during the study. In the end, the remaining 15 articles were included in the meta-analysis. The filtering flow chart is as follows (Figure 1).

### Research Characteristics

All 15 RCTs (24–38) enrolled 1,483 patients, including the experimental group (n = 754) and the control group (*n* = 729). In all studies, the control therapy was followed by conventional antihypertensive regimen, while in the observation group, the control therapy was combined with HQI intervention with a dose range of 20–60 mg/d. Detailed information about the included studies is provided in Table 1.

**Table 1:** Characteristics of the included studies.

**References**	**Sample size (T/C)**	**Sex M/F**		**Age (years)**		**Diagnosis standards**	**Treatment**	**Control**	**Duration of treatment**	**Outcomes**	**Adverse events**
		**T**	**C**	**T**	**C**						
Chen, (38)	90/90	54/36	51/39	43.25 ± 9.61	42.67 ± 9.72	CGMH (2005)	HQI (30 mL ivgtt qd) +control	Antihypertension therapy (no details) +Low protein, low salt and low fat diet (no details)	30 days	SBP, DBP, Scr, 24 h UTP, MDA, SOD, NO, NOS, Ep, PWVβ, AC	no mentioned
Dong, (29)	26/23	19/7	17/6	65 ± 3.2	64.3 ± 4.5	Diagnosis and Treatment of Nephropathy (Ye Rengao)	HQI (20 mL ivgtt qd) +control	Fosinopril Sodium (10–20 mg/d)	30 days	BUN, Scr	no mentioned
Guo, (32)	47/47	33/14	34/13	57.6 ± 9.3	57.8 ± 9.1	Essential hypertension with proteinuria	HQI (30 mL ivgtt qd) +control	Low protein, low salt and low fat diet (no details) +Irbesartan (150 mg/d)	4 weeks	SBP, DBP, BUN, Scr, Cys-c, 24 h UTP, NAG, α1-MG	no mentioned
Han, (24)	40/38	26/14	22/16	57.2 ± 3.5	57.8 ± 3.3	CGMH (2009)	HQI (40 mL ivgtt qd) +control	Irbesartan (150 mg/d)	30 days	SBP, DBP, hs-CRP, UAER, β2-MG, BUN, Ccr, TC, TG, HDL-C, LDL-C	no mentioned
He, (35)	50/46	40/10	38/8	74.2 (mean)		Essential hypertension with proteinuria	HQI (40 mL ivgtt qd) +control (without PGE1 injection)	Low protein, low salt and low fat diet (no details) +PGE1 injection 200 mg ivgtt qd	45 days	BUN, Scr, Ccr	no mentioned
Huang, (30)	45/45	54/36 (no details)		62.25 ± 8.52 (no details)		CGMH (2005)	HQI (30 mL ivgtt qd) +control	Irbesartan (150 mg/d)	4 weeks	Cys-c, mALB, β2-MG, NAG	no mentioned
Huang, (27)	63/63	41/22	40/23	58.36 ± 6.87	57.93 ± 6.97	Diagnosis and Treatment of Nephropathy (Ye Rengao)	HQI (30 mL ivgtt qd) +control	Irbesartan (150mg/d)	4 weeks	BUN, Scr, 24 h UTP, mALB, Cys-c, LP	Treatment group 2, Control group 4
Ji, (28)	54/40	36/18	23/17	56.3 (mean)	54.6 (mean)	CGMH (2000)	HQI (25 mL ivgtt qd) +control	Antihypertension therapy (no details)	30 days	mALB, β2-MG	no mentioned
Song, (31)	39/39	26/13	25/14	56.87 ± 11.55	55.26 ± 10.47	Nephrology (Wang Haiyan)	HQI (30 mL ivgtt qd) +control	Irbesartan (150 mg/d)	28 days	SBP, DBP, 24 h UTP, Scr, BUN	no mentioned
Tang, (25)	30/30	18/12	16/14	67.10 ± 9.94	62.96 ± 9.54	Nephrology (Wang Haiyan)	HQI (60 mL ivgtt qd) +control	Antihypertension therapy (no details) +Low protein, low salt and low fat diet (no details)	4 weeks	NAG, 24 h UTP, ET-1	no mentioned
Wu, (37)	23/23	15/8	16/7	54 ± 8	55 ± 9	CGMH (1999)	HQI (50 mL ivgtt qd) +control	Antihypertension therapy (no details) +Low protein, low salt and low fat diet (no details)	4 weeks	24 h UTP	no mentioned
Yang, (34)	80/78	51/29	50/28	no mentioned		CGMH (2014)	HQI (40 mL ivgtt qd) +control	Losartan Potassium (50 mg/d) +Hydrochlorothiazide (12.5 mg/d)	4 weeks	SBP, DBP, 24 h UTP, UAER	No adverse events
Zhao, (26)	56/56	31/25	32/24	62.1 ± 7.9	61.6 ± 8.2	Essential hypertension with renal damage	HQI (30 mL ivgtt qd) +control	Captopril (25 mg/d) + Low protein, low salt and low fat diet (no details)	30 days	Scr, BUN, Cys-c, 24 h UTP, NAG, α1-MG	no mentioned
Zhao, (33)	66/66	76/56 (no details)		57.33 ± 10.29 (no details)		Internal Medicine (Lu Zaiying)	HQI (20 mL ivgtt qd) +control	Irbesartan (150 mg/d) + Low protein, low salt and low fat diet (no details)	4 weeks	SBP, DBP, Scr, BUN, 24 h UTP, mAIB	no mentioned
Zhaoyj, (36)	45/45	23/22	22/23	67.6 ± 8.2	68.4 ± 8.7	CGMH (2005)	HQI (40 mL ivgtt qd) +control	Antihypertension therapy (no details) +Low protein, low salt and low fat diet (no details) +Quitting cigarettes and alcohol	4 weeks	SBP, DBP, UAER, β2-MG, TC, TG, HDL-C, LDL-C	No adverse events

*T, treatment group; C, control group; CGMH, Chinese guidelines for the management of hypertension; 24 h UTP, 24-h urinary total protein; AC, arterial compliance; BUN, blood urea nitrogen; Ccr, creatinine clearance rate; Cys-c, cystatin C; DBP, diastolic blood pressure; Ep, pressure-strain elasticty modulus; ET-1, endothelin-1; HDL-C, high density lipoprotein cholesterol; hs-CRP, high-sensitivity C-reactive protein; LDL-C, low density lipoprotein cholesterol; LP, lipoprotein; mAIB, microalbumin; MDA, malondialdehyde; NAG, N-acetyl-beta-D-glucosaminidase; NO, nitric oxide; NOS, nitric Oxide Synthase; PWVβ, pulse wave velocity β; SBP, systolic blood pressure; Scr, serum creatinine; SOD, superoxide dismutase; TC, total cholesterol; TG, triglycerides; UAER, urinary protein excretion rate; α1-MG, alpha-1- macroglobulin; β2-MG, beta-2-microglobulin*.

### Quality Assessment

**Figure 2:**
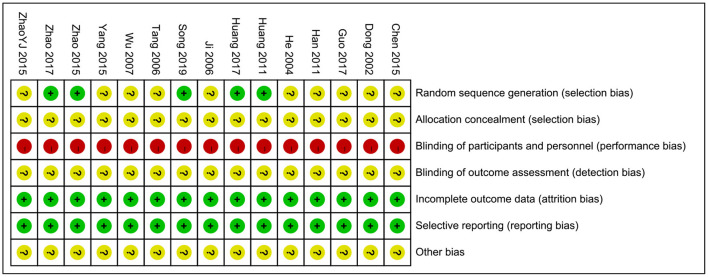
Risk of bias summary.

All of the included studies mentioned randomization, and only five of the trials described the randomization method used in their studies, while the others did not describe specific allocation techniques. None of the studies had procedures for hidden assignment and blinding. Quality assessment is shown in Figure 2.

### Results of Meta-Analysis

#### 24-H Urinary Total Protein (24 h UTP)

**Figure 3:**
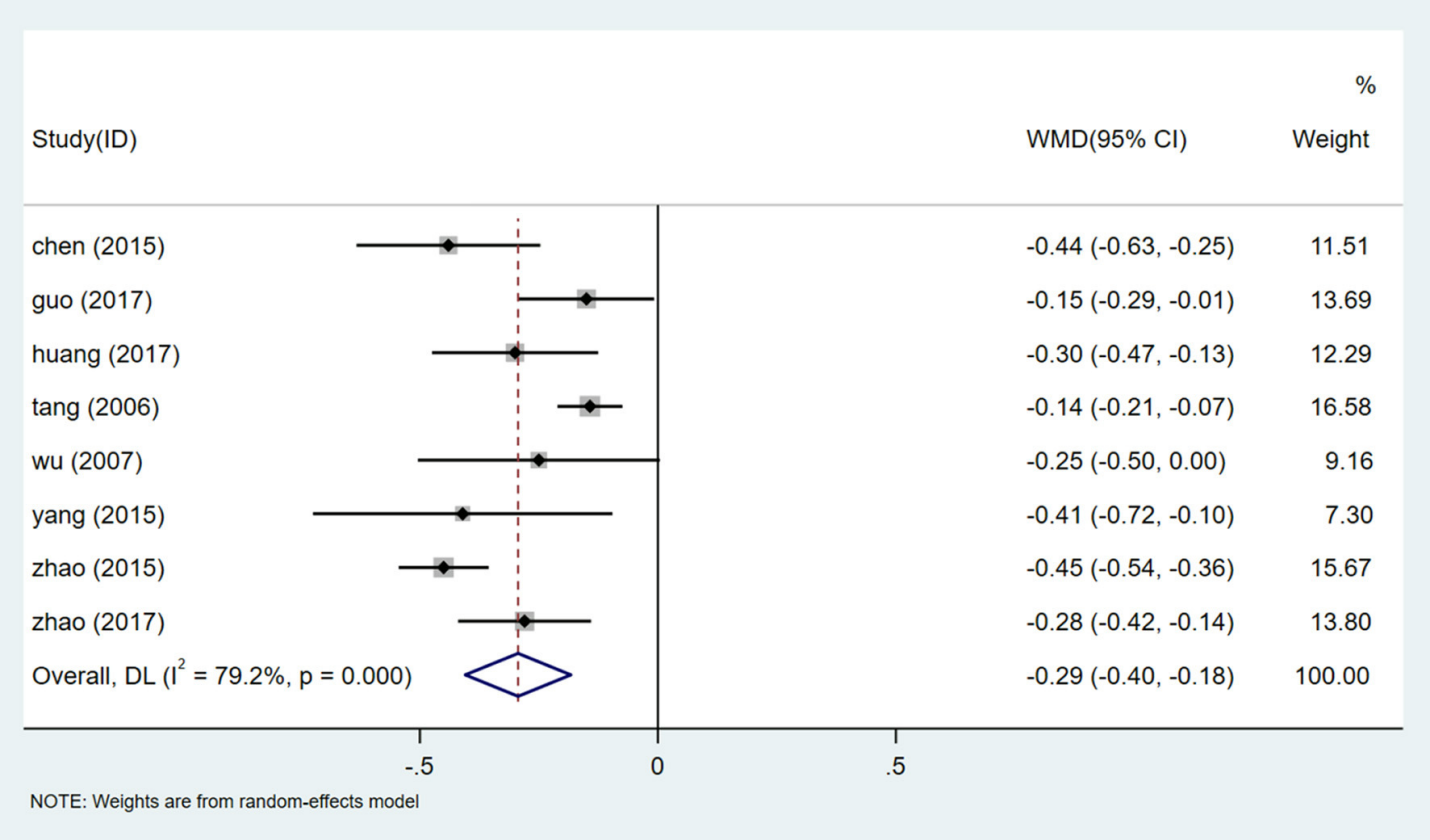
Forest plot of 24 h UTP.

There are 8 studies (25–27, 32–34, 37, 38) included a total of 908 participants reporting 24 h UTP. After heterogeneity was tested (I^2^ = 79.2%, *P* = 0.000, Supplementary Figure 1), a random effect model was used. A funnel chart analysis of 8 studies showed significant asymmetry, which may be related to publication bias or inclusion of low-quality studies (Supplementary Figure 2). Egger test (*P* = 0.372) (Supplementary Figure 3) was used to evaluate publication bias, and the results showed that there was no publication bias. The results of meta-analysis showed that the experimental group was superior to the control group in reducing 24h UTP [WMD = −0.29, 95% CI (−0.40, −0.18), *P* = 0.000] (Figure 3, Supplementary Figure 1). The difference was statistically significant, and patients with HQI combined with routine antihypertensive intervention had more significant efficacy in reducing 24 h UTP.

#### Sensitivity Analysis of 24 h UTP

We conducted a sensitivity analysis of 24 h UTP (Supplementary Figure 4). By excluding one inclusion study one by one, a meta-analysis of the remaining experiments was conducted to determine whether the results had changed significantly. Sensitivity analysis shows that the results of 24 h UTP are very similar and have relatively good stability.

#### Subgroup Analysis of 24 h UTP

**Figure 4:**
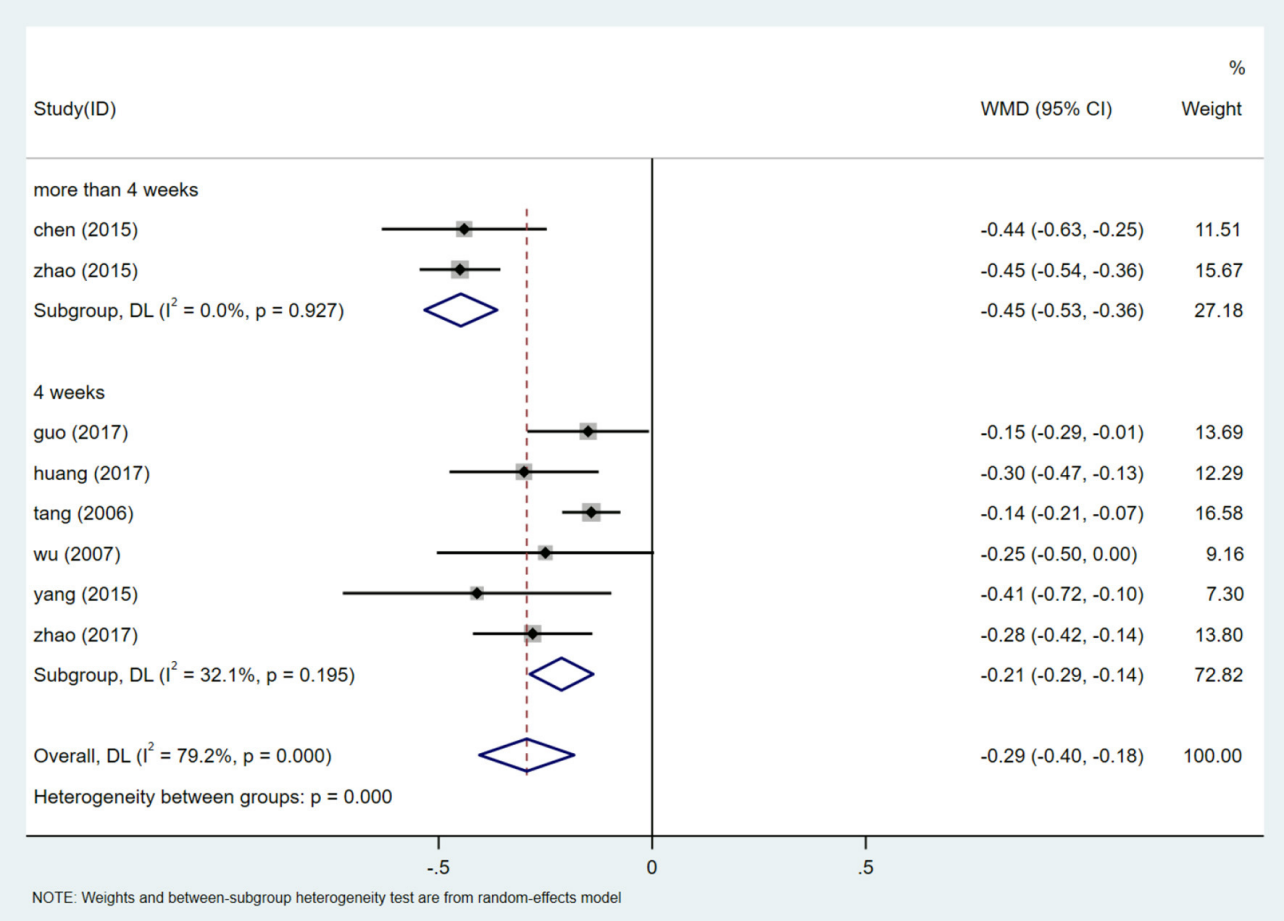
Subgroups analysis of 24 h UTP.

There is a high degree of heterogeneity in the evaluation of 24 h UTP. In order to determine the source of heterogeneity, we included the dose of HQI, antihypertensive regimen, course of treatment, and the year in which the study was published. Univariate Meta regression analysis was performed on the parameters of 8 studies (Supplementary Figure 5). The results show that the source of heterogeneity of HQI intervention in 24 h UTP may be related to the course of treatment (*P* = 0.001). The subgroup analysis was carried out based on the course of treatment. The results of meta-analysis showed that the heterogeneity was lower in the subgroup with a treatment cycle of 4 weeks [WMD = −0.212, 95% CI (−0.287, −0.138), *P* = 0.000, I^2^ = 0.0%], The results were statistically significant (Figure 4). Meta-analysis showed that the results were still statistically significant for subgroups with more than 4 weeks of treatment [WMD = −0.448, 95% CI (−0.287, −0.138), *P* = 0.000, I^2^ = 0.0%] (Supplementary Figure 6). Results of the subgroup analysis showed a statistically significant reduction in 24h UTP levels in studies that treated for more than 4 weeks compared with studies that treated for 4 weeks (*P* = 0.000). It is suggested that prolonging the treatment period of HQI may benefit patients with a reduction of 24 h UTP.

#### Microalbumin (mALB)

**Figure 5:**
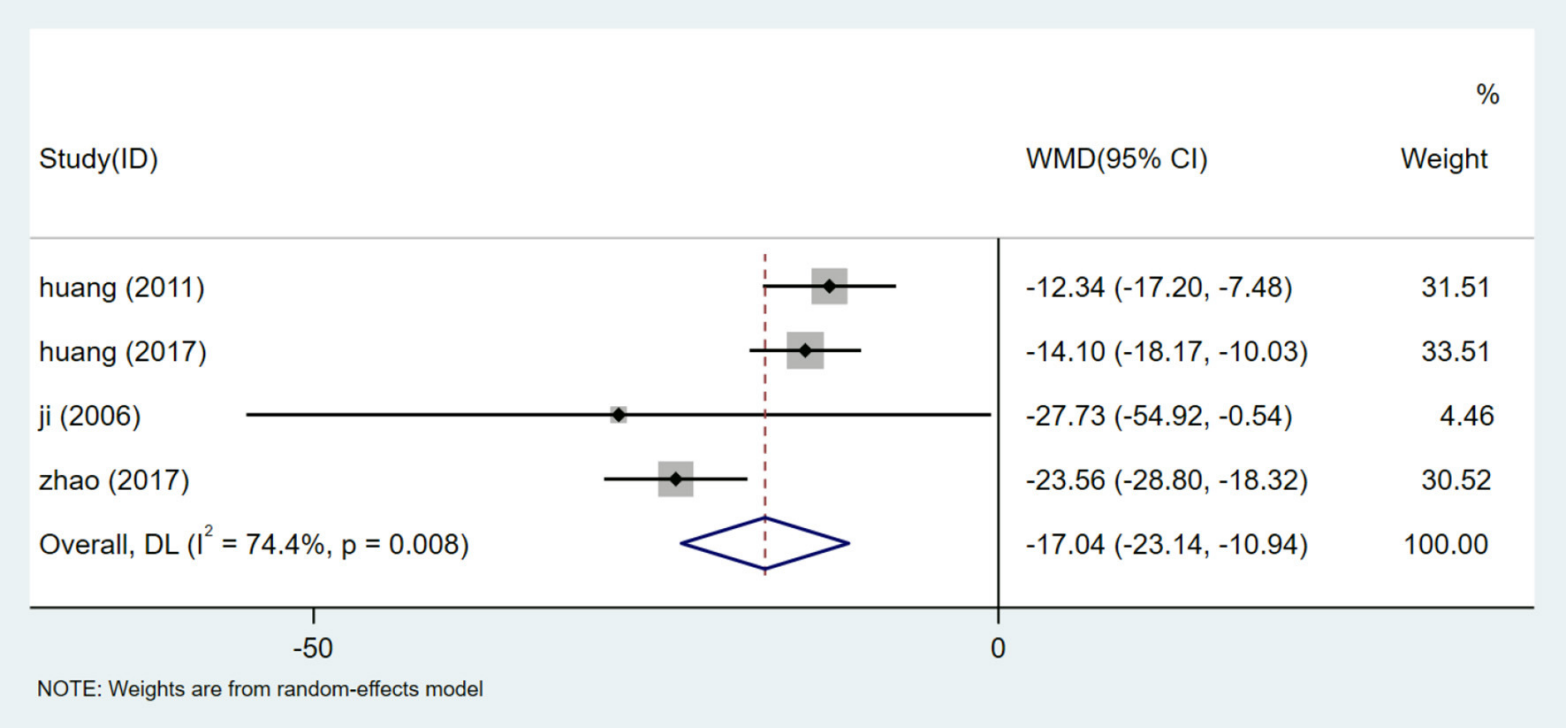
Forest plot of mALB.

**Figure 6:**
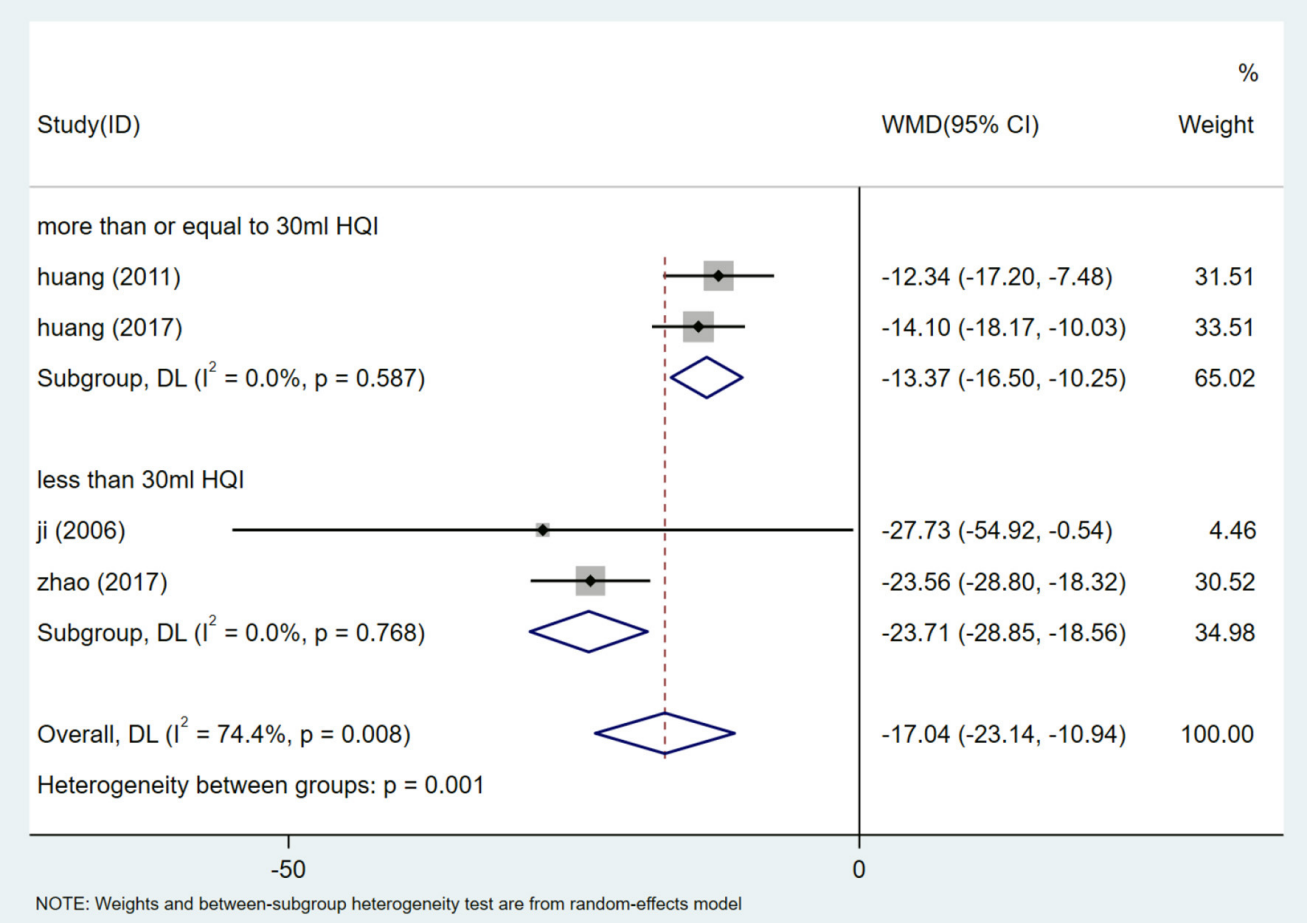
Subgroups analysis of mALB.

A total of 442 participants from 4 studies reported mALB levels (27, 28, 30, 33). After heterogeneity test (I^2^ = 74.4%, *P* = 0.008, Supplementary Figure 7), random effects model was used to summarize the data. Publication bias was assessed by Egger test (*P* = 0.629) (Supplementary Figure 8), and sensitivity analysis of mALB was performed (Supplementary Figure 9). The results showed that there was no significant publication bias, and meta-analysis was conducted to exclude the study at a time. The results of mALB were similar and relatively stable. The results of meta-analysis show that HQI combined with conventional antihypertensive regimen is more effective in reducing mALB [WMD = −17.04, 95% CI (−23.14, −10.94), *P* = 0.000] (Figure 5, Supplementary Figure 7). Due to the heterogeneity, different doses of HQI were used for subgroup analysis in our study. The effect of subgroup of not <30 mL/d HQI was used [WMD = −13.375, 95% CI (−16.497, −10.252), *P* = 0.000, I^2^ = 0.0%] is more significant than subgroup using HQI below 30 mL/d [WMD = −23.71, 95% CI (−28.85, −18.56), *P* = 0.000, I^2^ = 0.0%], the results were statistically significant (*P* = 0.001) (Figure 6, Supplementary Figure 10).

#### Serum Creatinine (Scr)

**Figure 7:**
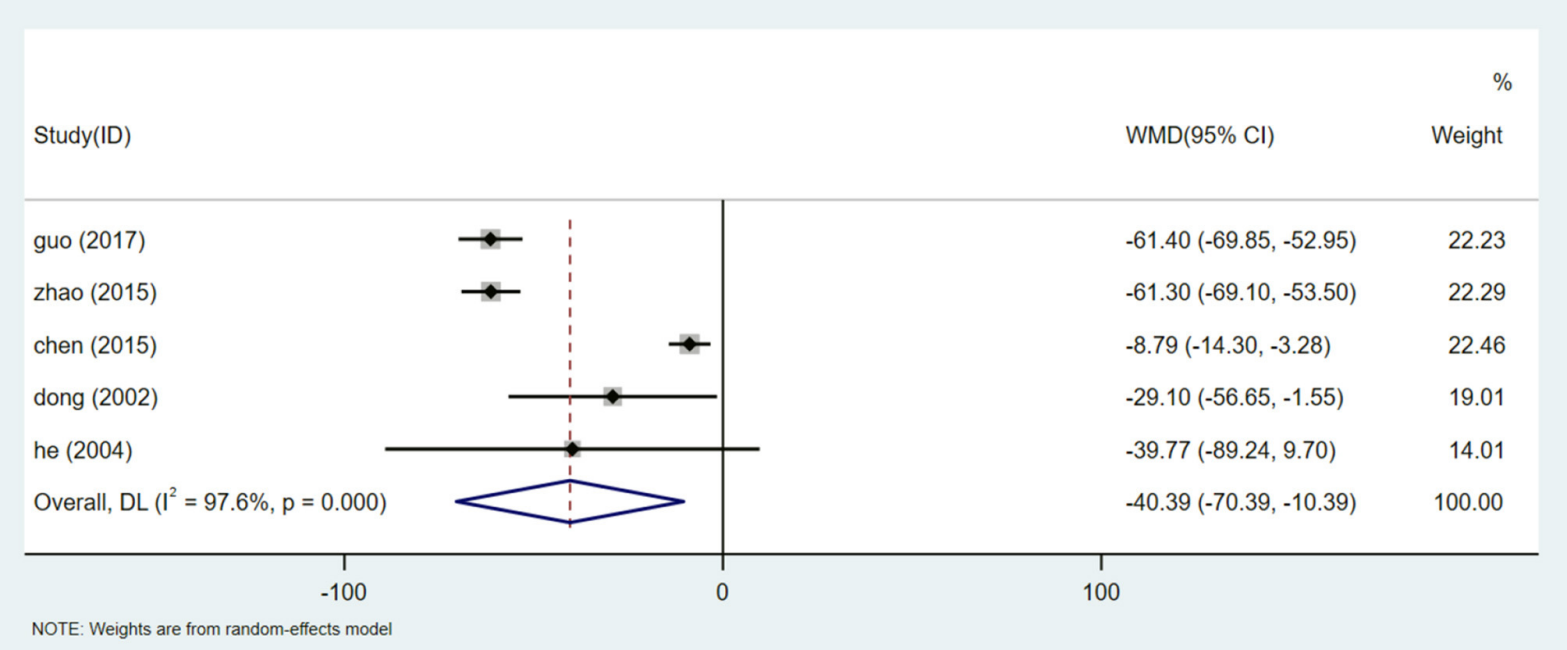
Forest plot of Scr.

**Figure 8:**
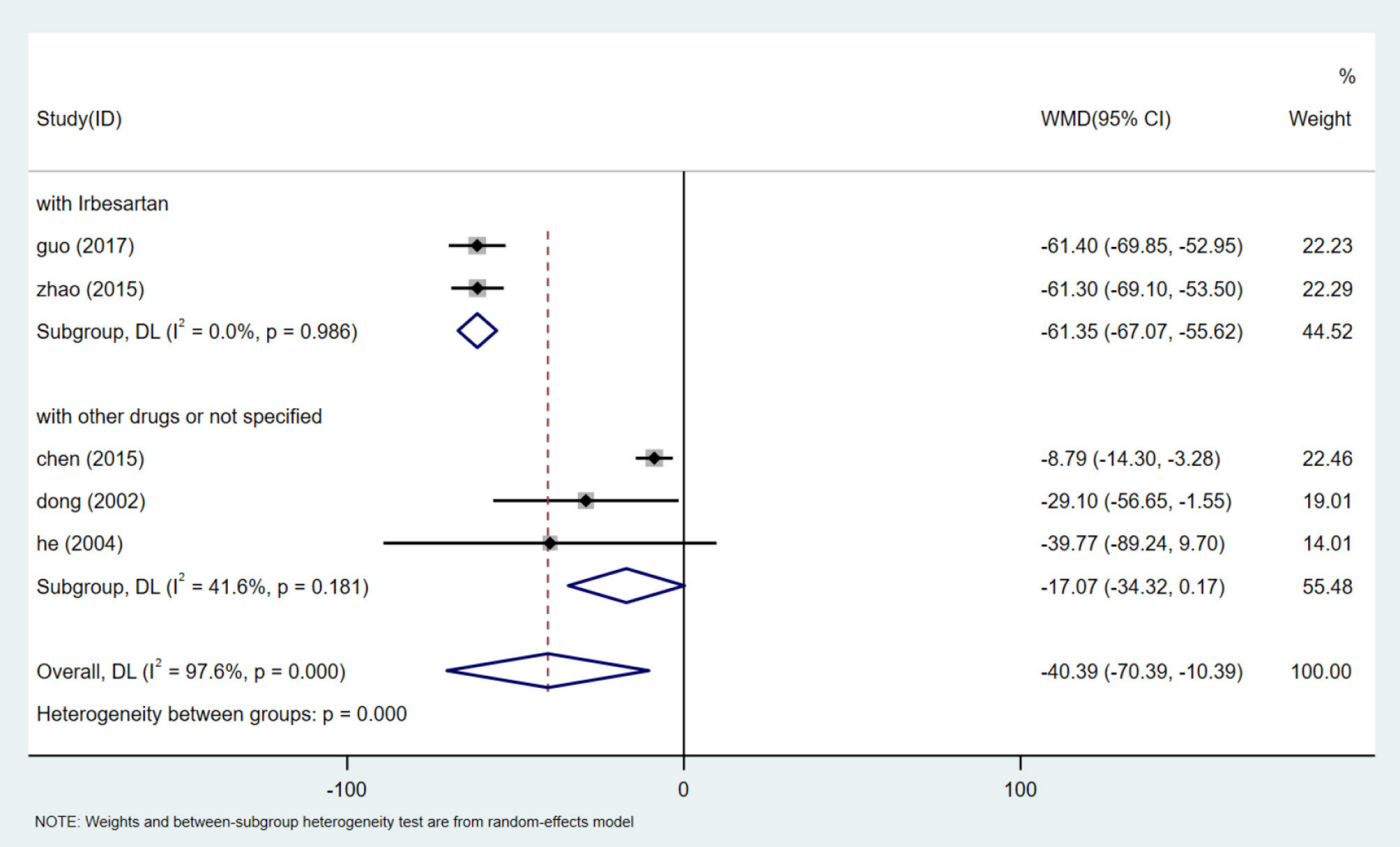
Subgroups analysis of Scr.

Five studies (26, 29, 32, 35, 38) reported Scr analysis, involving a total of 531 participants. After the heterogeneity test (I^2^ = 97.6%, *P* = 0.000, Supplementary Figure 11), the random effect model was used to evaluate the data. Egger test (*P* = 0.659) (Supplementary Figure 12) showed that there was no significant publication bias. Sensitivity analysis shows the stability of the results (Supplementary Figure 13). Meta-analysis showed that the Scr of patients receiving HQI combined with routine antihypertensive regimen was significantly lower than that of the control group [WMD = −40.39, 95% CI (−70.39, −10.39), *P* = 0.008] (Figure 7, Supplementary Figure 11). We also carried out a subgroup analysis based on the selection of conventional antihypertensive schemes. The results showed that HQI combined with irbesartan was effective in reducing Scr [WMD = −61.346, 95% CI (−67.075, −55.617), *P* = 0.000, I^2^ = 0.0%], In the subgroup of HQI combined with other antihypertensive drugs, the decreasing trend of Scr between the experimental group and the control group was not significant [WMD = −17.073, 95% CI (−34.315, 0.169), *P* = 0.052, I^2^ = 41.6%] (Figure 8, Supplementary Figure 14).

#### Systolic Blood Pressure (SBP)

**Figure 9:**
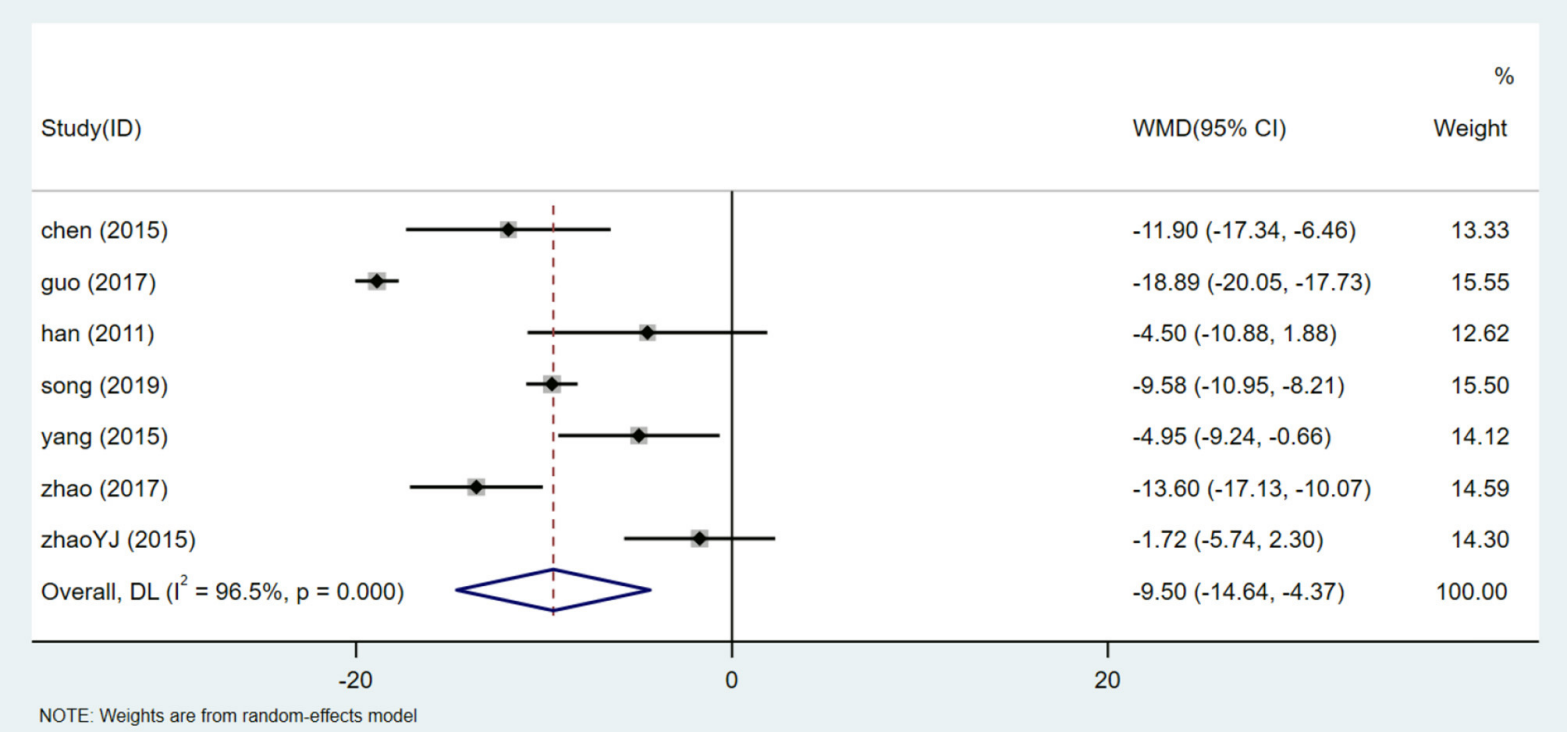
Forest plot of SBP.

**Figure 10:**
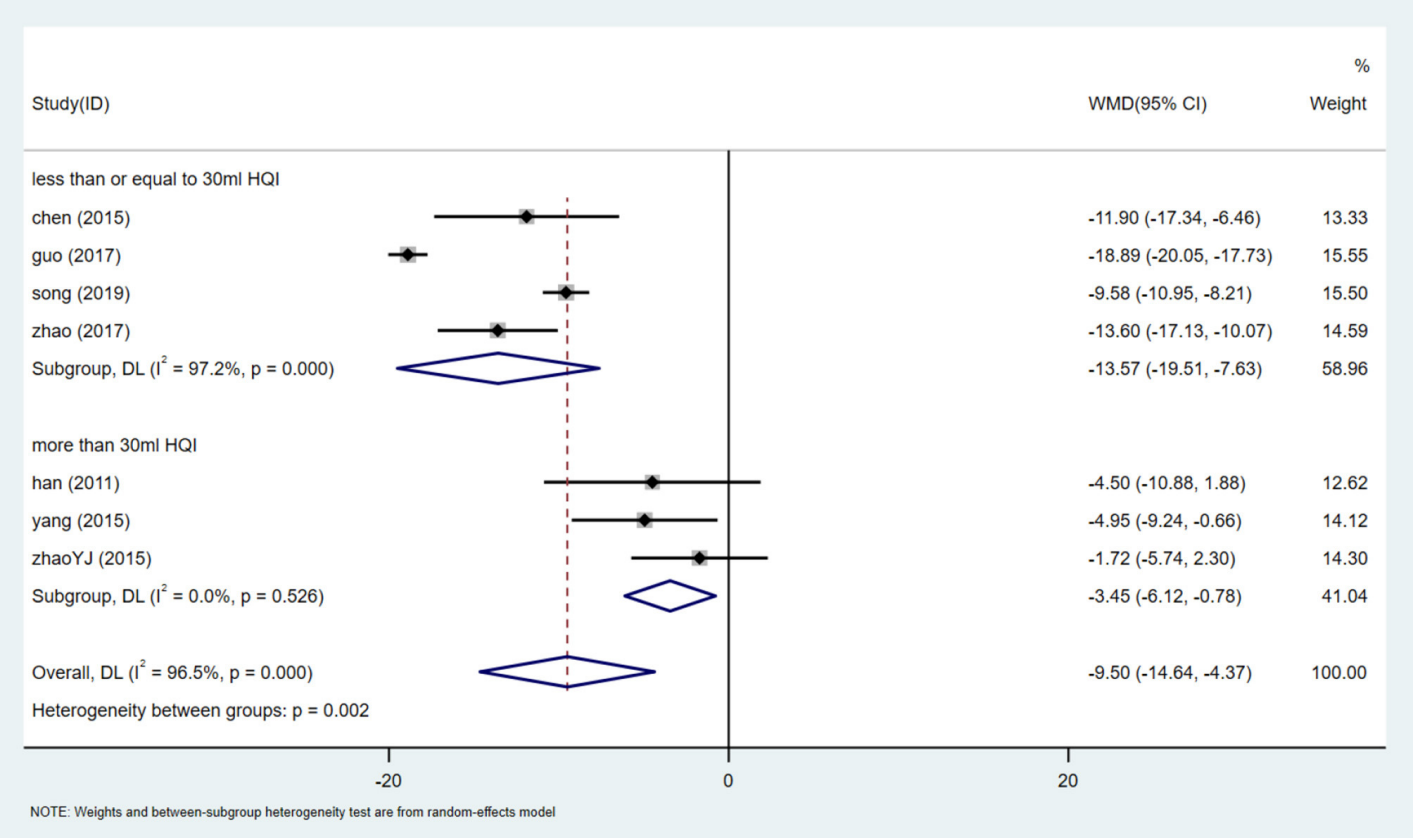
Subgroups analysis of SBP.

There are 7 studies (24, 31–34, 36, 38) involving 810 participants that reported SBP levels. The random effect model was used after heterogeneity test (I^2^ = 96.5%, *P* = 0.000, Supplementary Figure 15). We used sensitivity analysis to test the stability of the results (Supplementary Figure 16), and Egger test (*P* = 0.188) (Supplementary Figure 17) to evaluate the publication bias of SBP. Meta-analysis showed that patients treated with HQI combined with conventional antihypertensive regimen had better SBP management [WMD = −9.50, 95% CI (−14.64, −4.37), *P* = 0.000] (Figure 9, Supplementary Figure 15). The subgroup analysis based on the dose of HQI showed that there was a correlation between the reduction of SBP and the dose of HQI. The results showed that the heterogeneity decreased in the subgroup using more than 30mL HQI [WMD = −3.451, 95% CI (−14.642, −4.366), *P* = 0.011, I^2^ = 0.0%]. the subgroups using less than the dose of 30 mL HQI performed better [WMD = −13.570, 95% CI (−19.506, −7.633), *P* = 0.000, I^2^ = 97.2%] (Figure 10, Supplementary Figure 18), the results are statistically significant (*P* = 0.002). It is suggested that patients receiving dose intervention of no more than 30 mL HQI will benefit more than those who receive dose more than 30 mL HQI. However, the subgroup using <30 mL HQI still has high heterogeneity, suggesting that there are other sources of heterogeneity, which may be related to the low quality of the included study.

#### Diastolic Blood Pressure (DBP)

**Figure 11:**
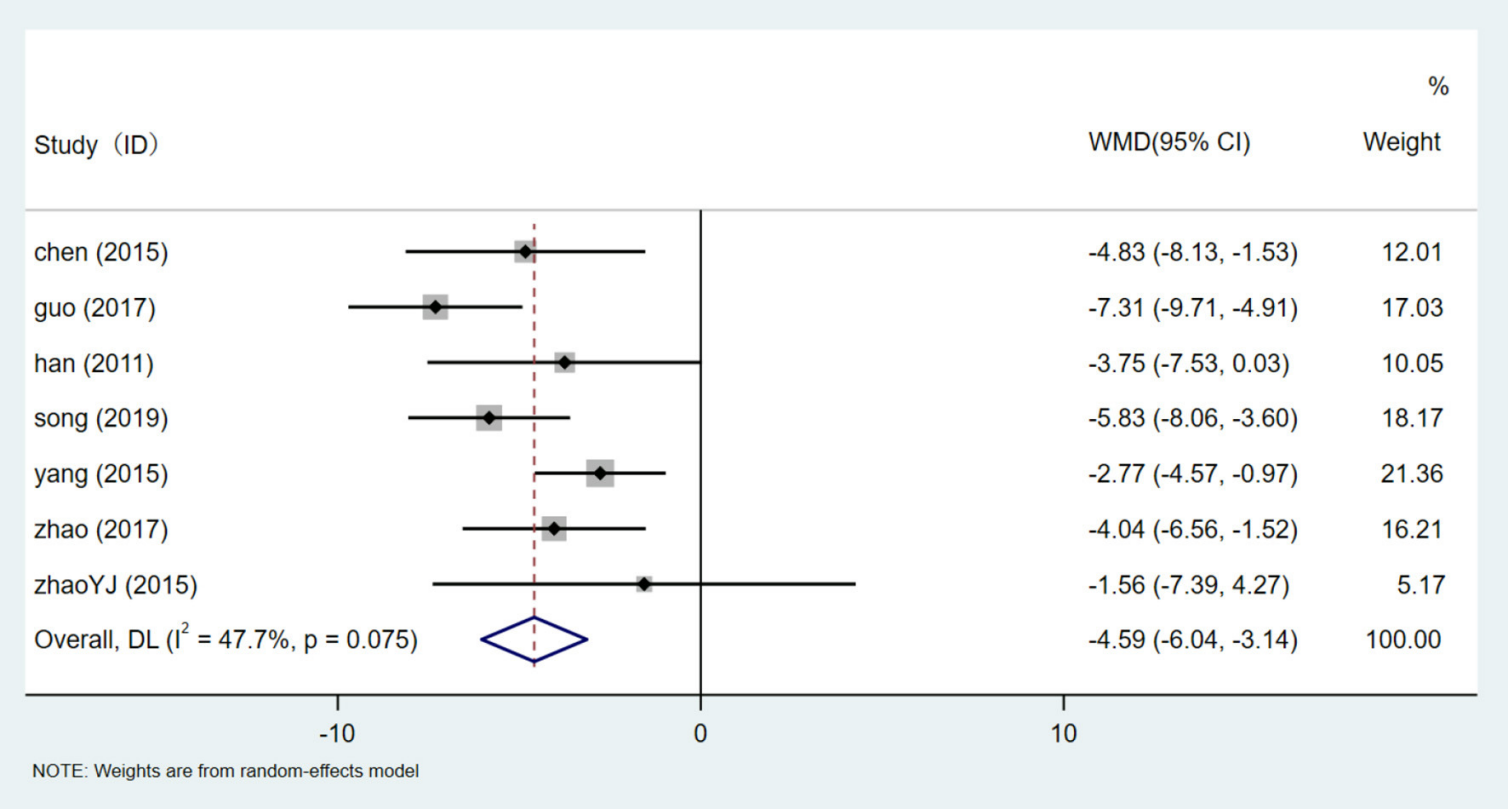
Forest plot of DBP.

**Figure 12:**
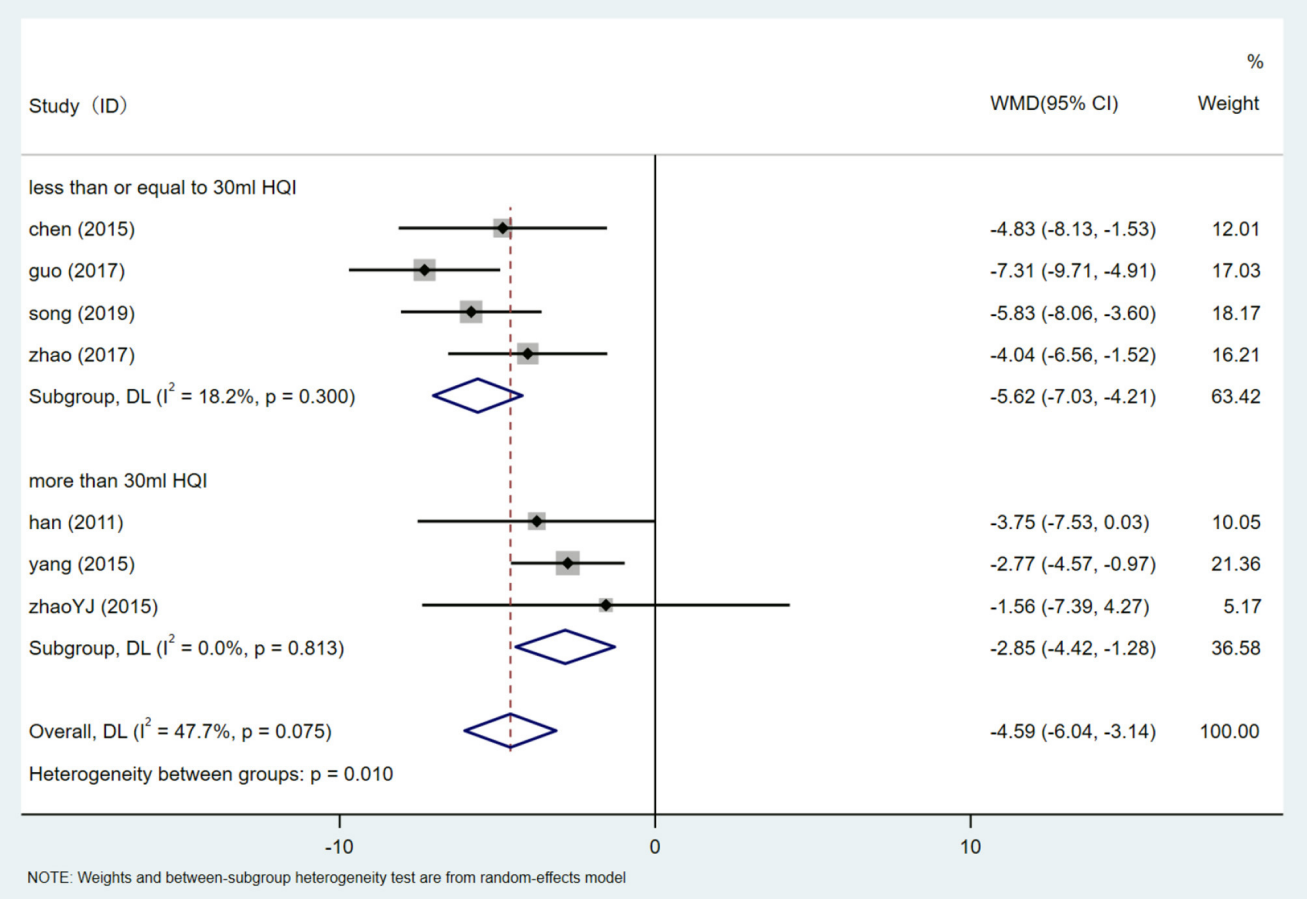
Subgroups analysis of DBP.

There are 7 studies (24, 31–34, 36, 38) that involved 810 participants assessed the level of DBP. After heterogeneity test (I^2^ = 47.7%, *P* = 0.075, Supplementary Figure 19), a fixed-effect model was used.Egger test (*P* = 0.900) (Supplementary Figure 20) was used to evaluate DBP publication bias. Sensitivity analysis showed that the results of DBP were relatively analogical, while the results of successive exclusion of one trial and reanalysis of the meta-analysis were relatively stable (Supplementary Figure 21). The results showed that HQI combined with conventional antihypertensive regimen was superior to the control group in reducing DBP level [WMD = −4.588, 95% CI (−6.036, −3.140), *P* = 0.000] (Figure 11, Supplementary Figure 19). Subgroup analysis based on HQI usage dose showed that subgroup using more than 30 ml HQI [WMD = −4.588, 95% CI (−6.036, −3.140), *P* = 0.000, I^2^ = 0.0%] and <30 mL HQI [WMD = −5.623, 95% CI (−7.033, −4.213), *P* = 0.000, I^2^ = 18.2%] (Figure 12, Supplementary Figure 22) both reduce heterogeneity, and the results were statistically significant (*P* = 0.010). There was a correlation between the decrease of DBP and the dose of HQI, and the results were consistent with the intervention of SBP with HQI combined with conventional antihypertensive regimen.

#### Cystatin C (Cys-c)

**Figure 13:**
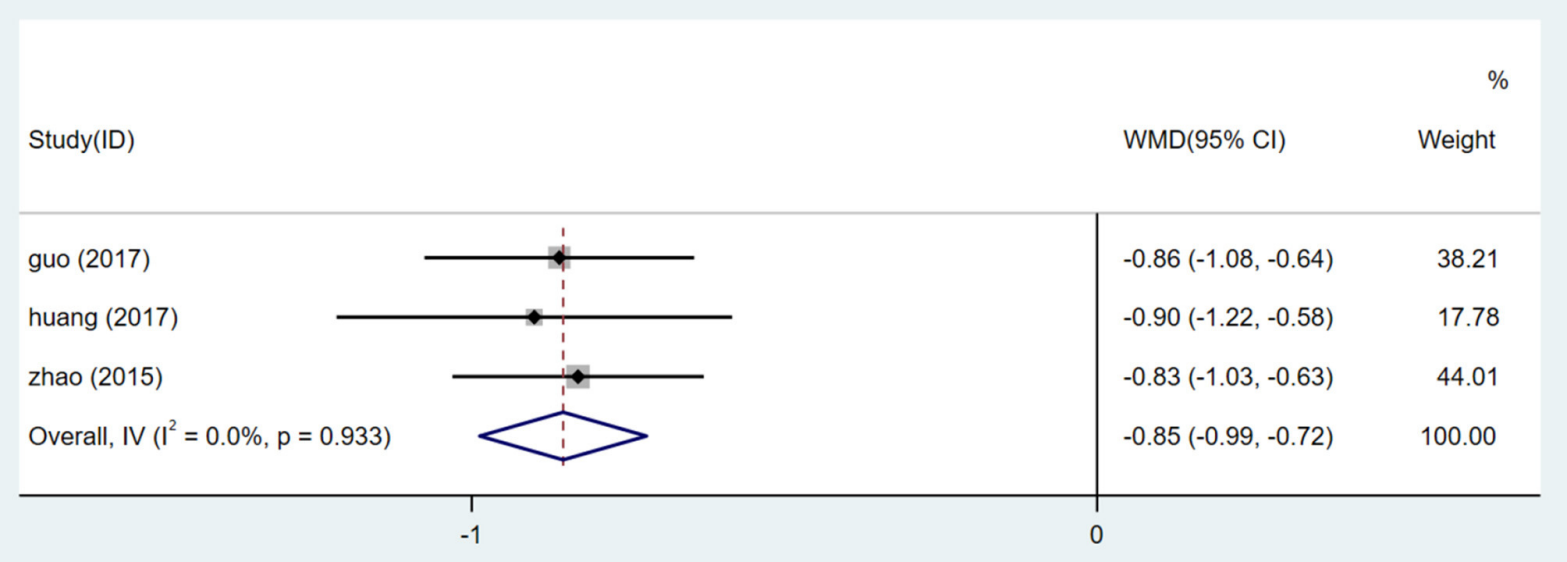
Forest plot of Cys-c.

Only four studies (26, 27, 30, 32) included Cys-c levels. After testing the heterogeneity (I^2^ = 76.9%, *P* = 0.005, Supplementary Figure 23), the random effect model was used. We performed an Egger test (*P* = 0.336) (Supplementary Figure 24) to assess publication bias. Considering the high degree of heterogeneity, sensitivity analysis was conducted on Cys-C data. By excluding one study one by one and re-meta-analysis of the other studies (Supplementary Figure 25), we found that the study from an article led to an increase in sensitivity. After elimination, meta-analysis was carried out. After heterogeneity test (I^2^ = 0.0%, *P* = 0.933, Supplementary Figure 26), a fixed effect model was used. Meta-analysis showed lower Cys-C in patients treated with HQI combined with conventional antihypertensive regimens [WMD = −0.854, 95% CI (−0.987, −0.721), *P* = 0.000] (Figure 13, Supplementary Figure 26).

#### Blood Urea Nitrogen (BUN)

**Figure 14:**
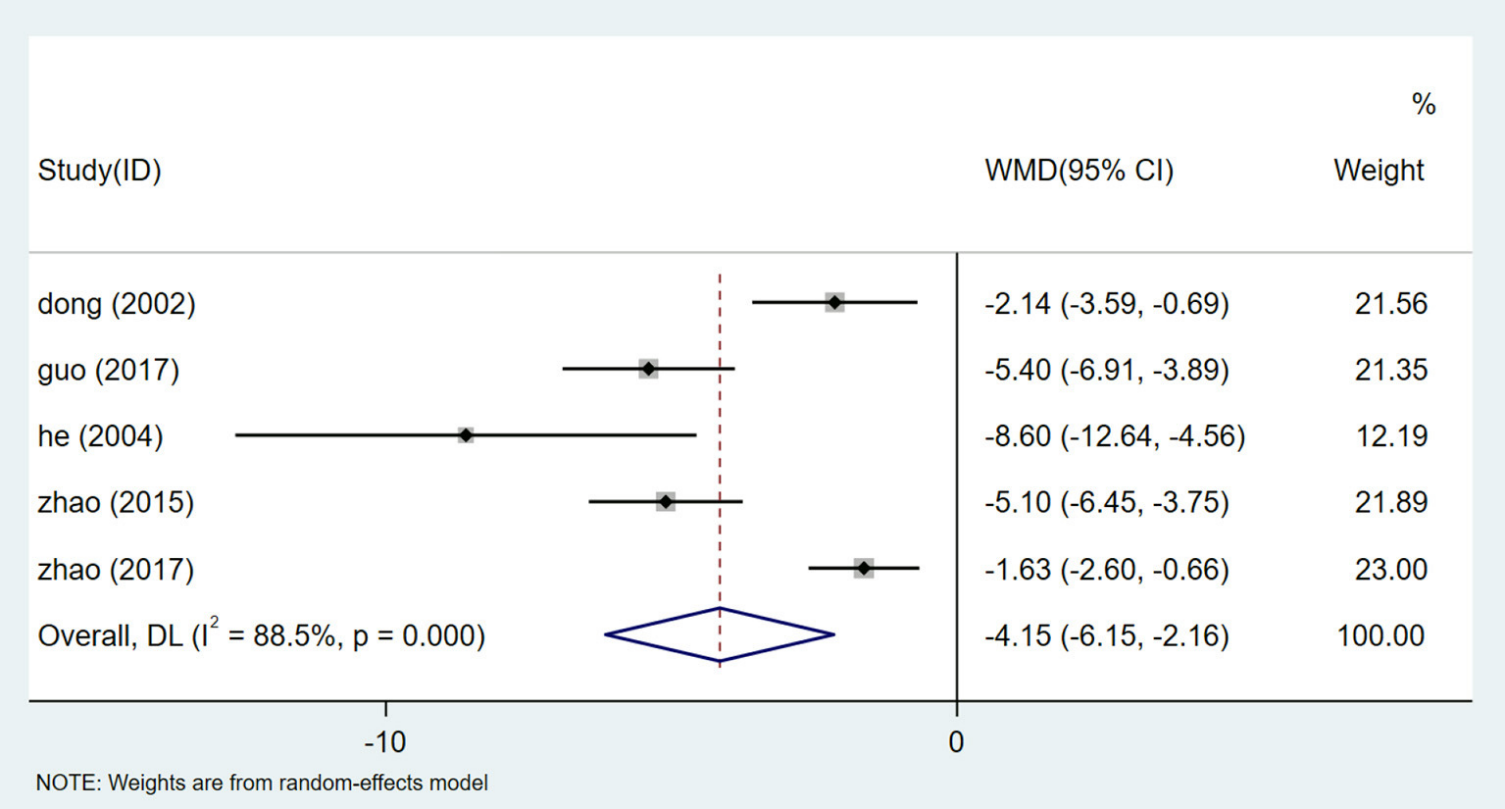
Forest plot of BUN.

There are 5 studies (26, 29, 32, 33, 35) that showed BUN results. After the heterogeneity test (I^2^ = 88.5%, *P* = 0.000, Supplementary Figure 27), the random effect model was used. The publication bias was assessed by Egger test (*P* = 0.191) (Supplementary Figure 28). High sensitivity was detected, and sensitivity analysis was used, and the results showed that the stability was high (Supplementary Figure 29). Meta-analysis showed that the therapeutic effect of HQI combined with conventional antihypertensive regimen was better than that of the control group [WMD = −4.155, 95% CI (−6.152, −2.157), *P* = 0.000] (Figure 14, Supplementary Figure 27).

## Discussion

The increase of blood pressure is closely related to the progress of CKD. The prevalence of CKD and ESRD caused by hypertension continues to rise worldwide. It is a challenge to identify biomarkers of early renal insufficiency due to the lack of unified criteria for the diagnosis of hypertensive nephropathy. Hypertension-related renal dysfunction can lead to an increase in serum creatinine (Scr) which eventually lead to irreversible kidney damage. Urinary protein can be used to evaluate hypertension-related kidney damage caused by glomerular disease, and urinary mALB is also considered as a potential marker of early renal dysfunction (9). Scr, urinary protein and urinary microprotein have become important indicators to evaluate the protective effect of antihypertensive drugs on kidney in clinical trials, and that is also the reason why we take them as the main evaluation indexes (39–41). Cys-c is another biomarker that reflects the level of renal function. Studies have shown that Cys-c-based eGFR is superior to Scr-based eGFR in predicting the risk of cardiovascular disease and chronic kidney disease (42). It has important reference value in evaluating the prognosis of hypertensive nephropathy. BUN is usually considered as an important serum marker to evaluate renal function. BUN plays an important role in the diagnosis of renal disease, prediction of cardiovascular events caused by acute heart failure and prognosis in patients with acute myocardial infarction (43, 44).

The incidence of hypertension and hypertension-related chronic kidney disease is increasing year by year, which is an independent risk factor for the morbidity and mortality of cardiovascular disease. Hypertension is not only the cause of kidney disease, but also the result of kidney disease, so hypertension nephropathy is bound to become a heavy burden in the field of public health and medical insurance. Strict antihypertensive treatment and measures to minimize proteinuria can significantly improve the prognosis of patients with hypertensive nephropathy (45–47). Although the pathogenesis of hypertensive nephropathy is unclear, current evidence suggest that RAAS is associated with hypertension and kidney disease (7, 48). The guidelines recommend that RAAS-inhibitor durgs are the first-line choice for hypertensive patients complicated with CKD (8). Unfortunately, the management of blood pressure in patients with hypertensive nephropathy is very difficult. A data from a trial of IDNT showed that only 30% of patients met the goal of systolic blood pressure management (49). Predictably, the proportion of patients whose blood pressure are effectively controlled in clinical practice is much lower than the reported data. Therefore, it is critical to explore other effective treatments for patients with hypertensive nephropathy.

HQI is the extract of Astragalus membranaceus (Fisch.) Bunge. More and more evidence show that HQI can reduce hypertension and protect kidney. Modern pharmacological studies have proved that HQI has a variety of active components. HQI has obvious advantages in the treatment of hypertensive nephropathy. Some pharmacological experiments on the efficacy and mechanism of HQI in treating hypertensive nephropathy have suggested that it plays an important role in improving renal perfusion, managing blood pressure and delaying renal function progression. (1) Improving hemodynamics. Studies have shown that HQI can affect the expression of renal vasoactive substances, improve renal hemodynamics and reduce tissue ischemia and hypoxia (50). (2) Anti-renal fibrosis. Astragalus polysaccharides can reduce the expression of Transforming Growth Factor β (TGF- β) and connective Tissue Growth Factor (CTGF) in renal tissue, reduce the excessive deposition of extracellular matrix and improve interrenal fibrosis (51, 52). (3) Regulation of vascular cell migration and proliferation. Astragaloside can regulate the effect of Protein Kinase D1 (PKD1)-Histone Deacetylase 5 (HDAC5)-Vascular Endothelial Growth Factor (VEGF) on vascular growth, migration and differentiation in rats (53). (4) Blood pressure management. Astragaloside IV can improve the diastolic and systolic function of the heart and has a two-way regulation, thus playing a role in the regulation of blood pressure (54). (5) Protective effect on myocardial ischemia injury. By increasing the content of cAMP, HQI can fully play the role of positive myodynamia, improve the stability of cardiomyocytes, reduce myocardial oxygen consumption, avoid ischemia-reperfusion injury, and improve the ability of myocardial antioxidation at the same time (55). These studies support the positive effects of HQI in relieving hypertensive nephropathy, but the efficacy of HQI combined with antihypertensive drugs in hypertensive nephropathy remains to be further reviewed and analyzed.

This meta-analysis included 15 RCTs and involved 1,483 patients to evaluating the relationship between HQI combined with antihypertensive agents and the use of antihypertensive agents alone in the treatment of hypertensive nephropathy. Based on the analysis of available data, we found that the efficacy of HQI combined with antihypertensive drugs is better than antihypertensive drugs used alone in the treatment of hypertensive nephropathy. The results showed that all of the involved patients improved from baseline, but the patients who received HQI combined with conventional antihypertensive therapies were more effective in improving 24 h UTP, mALB, Scr, SBP, DBP, Cys-c and BUN than those who only received conventional antihypertensive therapies. Compared with using antihypertensive drugs alone, patients with hypertensive nephropathy treated with HQI have significant advantages in reducing 24 h UTP. Although the included studies showed a high degree of heterogeneity, we conducted a subgroup analysis based on different courses of treatment. We observed the benefit of prolonging the course of HQI combination therapy in all subgroups with low heterogeneity. HQI combination treatment showed statistically significant advantages in the intervention of mALB, SBP and DBP. Interestingly, in the subgroup analysis based on the dosage of HQI, we observed that the intervention of antihypertensive drugs combined with not exceeding 30 mL HQI in hypertensive nephropathy was more effective in reducing mALB, SBP and DBP than that of antihypertensive drugs combined with more than 30 mL HQI, and the difference was statistically significant. We believe that the combined treatment dose of 30 mL HQI is a key point, which can achieve the goal of controlling mALB, SBP and DBP, and the use of large doses of HQI will not increase the benefits of patients. Therefore, excessive HQI combination therapy may be unreasonable. Considering that the methodological quality of these randomized controlled trials is low and the number of cases included is relatively small, the reliability of this conclusion needs to be further tested by prospective studies. In terms of Scr level, HQI combined with antihypertensive drugs was more effective than antihypertensive drugs alone. Subgroup analysis showed that HQI combined with irbesartan was superior to HQI combined with other antihypertensive drugs in the treatment of Scr. However, in view of the ambiguity of the description of other types of antihypertensive drugs in the included study, some studies lack specific and detailed drug regimen, and their reliability needs to be further confirmed, and this finding should be carefully interpreted.

### Limitations

Several limiting factors should be taken into account in this study. First of all, all the included studies were published in Chinese journals, which may lead to potential ethnic and geographical bias. Secondly, the methodological quality of the included randomized controlled trials is generally low, and all studies claim to be random, but only 5 studies mention the specific information generated by the sequence, so the so-called randomization method is questionable. Third, blinding is an important way to prevent research results from being affected by placebo effects or observer biases. All studies have no information about hidden allocation and participant blindness, which may lead to potential selection biases. Fourth, safety is the basic principle for the provision of herbal products for adjuvant therapy. Twelve studies have not reported adverse drug events and adverse reactions, and there is not enough evidence to conclude safety because some of the active components of the herbal may be unstable. Finally, there are no clear criteria for the diagnosis of hypertensive nephropathy, and the different diagnostic criteria used in the study may affect the reliability of the results.

## Conclusion

The results of this meta-analysis show that the combination of HQI and antihypertensive drugs is more significant in improving the related indexes of patients with hypertensive nephropathy than using antihypertensive drugs alone, and a evidence dose of HQI (no more than 30 mL) may benefit more. HQI combined with antihypertensive drugs has significant advantages in blood pressure management and renal function improvement in patients with hypertensive nephropathy. However, the quality of the methodology is low and the number of samples is small, the results need to be confirmed by more stringent randomized controlled trials.

## Data Availability Statement

The original contributions presented in the study are included in the article/Supplementary Material, further inquiries can be directed to the corresponding author.

## Author Contributions

ZX and CL conceived the study. ZX and LQ evaluated the included studies, conducted data extraction, and drafted manuscripts. RN checked the data with LQ. YY analyze the data. CL and XL supervised all aspects of the study. All authors contributed to the article and approved the submitted version.

## Funding

This work was supported by the National Natural Science Foundation of China (No. 81973762) and the Postgraduate Research & Practice Innovation Program of Jiangsu Province (KYCX20_1477 and KYCX20_1455).

## Conflict of Interest

The authors declare that the research was conducted in the absence of any commercial or financial relationships that could be construed as a potential conflict of interest.

## Publisher's Note

All claims expressed in this article are solely those of the authors and do not necessarily represent those of their affiliated organizations, or those of the publisher, the editors and the reviewers. Any product that may be evaluated in this article, or claim that may be made by its manufacturer, is not guaranteed or endorsed by the publisher.

## Supplementary Material

Supplementary File 1 Details of each study.

Supplementary File 2 Search Strategy.

## References

[1] Lawes CM Vander HS Rodgers A. Global burden of blood-pressure-related disease, 2001. Lancet. (2008) 371:1513–8. 10.1016/S0140-6736(08)60655-8 18456100

[2] Rigo D Orias M. Hypertension and kidney disease progression. Clin Nephrol. (2020) 93:103–7. 10.5414/CNP92S118 31549630

[3] Hart PD Bakris GL. Hypertensive nephropathy: prevention and treatment recommendations. Expert Opin Pharmaco. (2010) 11:2675–86. 10.1517/14656566.2010.485612 20718588

[4] Bidani AK Polichnowski AJ Loutzenhiser R Griffin KA. Renal microvascular dysfunction, hypertension and CKD progression. Curr Opin Nephrol Hypertens. (2013) 22:1–9. 10.1097/MNH.0b013e32835b36c1 23132368 PMC3942995

[5] Udani S Lazich I Bakris GL. Epidemiology of hypertensive kidney disease. Nat Rev Nephrol. (2011) 7:11–21. 10.1038/nrneph.2010.154 21079654

[6] Bicescu G. Epidemiology of hypertensive kidney disease: diagnosis. Maedica (Bucur). (2010) 5:309–10. 21977180 PMC3152845

[7] Xu J Zhang C Shi X Li J Liu M Jiang W . Efficacy and Safety of Sodium Tanshinone IIA Sulfonate Injection on Hypertensive Nephropathy: A Systematic Review and Meta-Analysis. Front Pharmacol. (2019) 10:1542. 10.3389/fphar.2019.01542 31920681 PMC6937217

[8] Whelton PK Carey RM Aronow WS Casey DE Collins KJ Dennison Himmelfarb C . 2017 ACC/AHA/AAPA/ABC/ACPM/AGS/APhA/ASH/ASPC/NMA/PCNA Guideline for the Prevention, Detection, Evaluation, and Management of High Blood Pressure in Adults: Executive Summary: A Report of the American College of Cardiology/American Heart Association Task Force on Clinical Practice Guidelines. Circulation. (2018) 138:e426–83. 30354655 10.1161/CIR.0000000000000597

[9] Bakris GL. Implications of Albuminuria on Kidney Disease Progression. The journal of clinical hypertension (Greenwich, Conn.). (2004) 6(11 Suppl 3):18–22. 10.1111/j.1524-6175.2004.04065.x 15538107 PMC8109517

[10] Khosla N Sarafidis PA Bakris GL. Microalbuminuria. Clin Lab Med. (2006) 26:635–53. 10.1016/j.cll.2006.06.005 16938588

[11] Atkins RC Briganti EM Lewis JB Hunsicker LG Braden G Champion DCP . Proteinuria reduction and progression to renal failure in patients with type 2 diabetes mellitus and overt nephropathy. Am J Kidney Dis. (2005) 45:281–7. 10.1053/j.ajkd.2004.10.019 15685505

[12] de Zeeuw D Remuzzi G Parving HH Keane WF Zhang Z Shahinfar S . Proteinuria, a target for renoprotection in patients with type 2 diabetic nephropathy: lessons from RENAAL. Kidney Int. (2004) 65:2309–20. 10.1111/j.1523-1755.2004.00653.x 15149345

[13] Huang KC Su YC Sun MF Huang ST. Chinese Herbal Medicine Improves the Long-Term Survival Rate of Patients With Chronic Kidney Disease in Taiwan: A Nationwide Retrospective Population-Based Cohort Study. Front Pharmacol. (2018) 9:1117. 10.3389/fphar.2018.01117 30327604 PMC6174207

[14] Li Y Yan S Qian L Wu L Zheng Y Fang Z. Danhong Injection for the Treatment of Hypertensive Nephropathy: A Systematic Review and Meta-Analysis. Front Pharmacol. (2020) 11:909. 10.3389/fphar.2020.00909 32636745 PMC7316888

[15] Wu L Liu M Fang Z. Combined Therapy of Hypertensive Nephropathy with Breviscapine Injection and Antihypertensive Drugs: A Systematic Review and a Meta-Analysis. Evid Based Complement Alternat Med. (2018) 2018:2958717. 10.1155/2018/2958717 30671127 PMC6317107

[16] Yu H. Analysis of Chemical Components in Huangqi Injection Based on Ultra Performance Liquid Chromatography-Quadrupole-Time-of-Flight Mass Spectrometry. World Trad Chin Med. (2019) 14:809–17.

[17] Jing L Zhong-Zhen Z Hu-Biao C. Review of Astragali Radix. Chin Herb Med. (2011) 3:90–105.

[18] Peng Z. Analysis of therapeutic effect of Huangqi injection on 80 cases of acute and chronic nephritis. Chinese Foreign Med. (2011) 30:28.

[19] Ni Z. Effects of astragaloside on the secretion of stroma and expression of β1 integrin in human mesangial cells. Chin J Nephrol. (2000) 5:303–7.

[20] Gao W. Research progress in clinical application of Huangqi injection. J Chengde Med Coll. (2014) 31:129–31.

[21] Ma J. Study on arginine vasopressin, V_2 receptor and aquaporin-2 in rats with nephrotic syndrome and the therapeutic effect of astragalus. Journal of Nephrology and Dialysis and Kidney Transplantation. (1999) 4:315–8.

[22] Guo X. Research progress on pharmacological effects and clinical application of Huangqi injection. Chin Pharm. (2015) 26:3018–21.29772932

[23] Moher D Liberati A Tetzlaff J Altman DG. Preferred reporting items for systematic reviews and meta-analyses: the PRISMA statement. PLoS Med. (2009) 6:e1000097. 10.1371/journal.pmed.1000097 19621072 PMC2707599

[24] Han X. The effect of irbesartan combined with Huangqi injection on renal damage in patients with essential hypertension. Hebei Trad Chin Med. (2011) 33:1505–6.

[25] Tang G. The Protective Effect of Astragalus on the Early Renal Damage of Hypertension. Mod J Integr Trad Chin West Med. (2006) 1:26–27.

[26] Zhao Y. Effects of astragalus combined with irbesartan on renal function and urine protein in patients with hypertensive renal impairment. Hainan Med. (2015) 26:1028–30.

[27] Huang L. Study on the Efficacy and Mechanism of Astragalus Combined with Irbesartan in Treating Hypertensive Nephropathy. Mod Med Health. (2017) 33:3456–8.

[28] Ji Y. Effect of Huangqi Injection on Kidney Injury of Hypertension. Chin Med Emerg. (2006) 11:1237–8.

[29] Dong G. Huangqi Injection and Fosinopril Sodium in the Treatment of Hypertensive Renal Damage. J Hubei Coll Trad Chin Med. (2002) 2:46.

[30] Huang X. Preventive and therapeutic effects of Huangqi injection combined with irbesartan on early renal damage of hypertension. Shaanxi Med J. (2011) 40:1663–4.

[31] Song P. The effect of huangqi injection combined with irbesartan in the treatment of hypertensive nephropathy. J Univers Health. (2019) 21:278.

[32] Guo J. Analysis of the effect and mechanism of Huangqi injection combined with irbesartan in the treatment of hypertensive nephropathy. China Contemp Med. (2017) 24:76–8.

[33] Zhao F. Study on the curative effect of Huangqi injection combined with irbesartan in the treatment of hypertensive nephropathy. Shaanxi Trad Chin Med. (2017) 38:51–2.

[34] Yang J. Clinical Observation of Huangqi Injection Combined with Hyzaar in the Treatment of Essential Hypertension Complicated with Proteinuria. TCM Clin Res. (2015) 7:29–30.

[35] He X. Effect of Huangqi Injection on Kidney Injury of Elderly Patients with Hypertension. J Pract Med. (2004) 8:712–3.

[36] Zhaoyj Y. Clinical Observation of Huangqi Injection in Treating Renal Damage of Primary Hypertension. Chin J Evid Based Cardiovasc Med. (2015) 7:248–50.

[37] Wu X. Observation on Curative Effect of Huangqi Injection in Treating Primary Hypertension with Renal Damage. Prim Med Forum. (2007) 16:717–8.

[38] Chen X. Effect of Huangqi Injection on Antioxidant Capacity and Vascular Elasticity in Patients with Renal Hypertension. J Hainan Med Coll. (2015) 21:375–7.

[39] Yan L Ma J Guo X Tang J Zhang J Lu Z . Urinary albumin excretion and prevalence of microalbuminuria in a general Chinese population: a cross-sectional study. BMC Nephrol. (2014) 15:165. 10.1186/1471-2369-15-165 25308236 PMC4209030

[40] Kalaitzidis RG Bakris GL. Serum creatinine vs. albuminuria as biomarkers for the estimation of cardiovascular risk. Curr Vasc Pharmacol. (2010) 8:604–11. 10.2174/157016110792006914 20507275

[41] Bakris GL. Clinical importance of microalbuminuria in diabetes and hypertension. Curr Hypertens Rep. (2004) 6:352–6. 10.1007/s11906-004-0053-1 15341686

[42] Shlipak MG Matsushita K Arnlov J Inker LA Katz R Polkinghorne KR . Cystatin C versus creatinine in determining risk based on kidney function. N Engl J Med. (2013) 369:932–43. 10.1056/NEJMoa1214234 24004120 PMC3993094

[43] Wei D Ge M. The spatial distribution of BUN reference values of Chinese healthy adults: a cross-section study. Int J Biometeorol. (2018) 62:2099–107. 10.1007/s00484-018-1585-4 30368678

[44] Ke Z. The value of serum creatinine, cystatin-C, urea nitrogen and urine β_2-microglobulin in early renal injury of type 2 diabetes. China Medical Herald. (2013) 10:94–6.

[45] Hohenstein K Watschinger B. Hypertension and the kidney. Wien Med Wochenschr. (2008) 158:359–64. 10.1007/s10354-008-0558-3 18677585

[46] Ravera M Re M Deferrari L Vettoretti S Deferrari G. Importance of blood pressure control in chronic kidney disease. J Am Soc Nephrol. (2006) 17 (4 Suppl 2): S98–103. 10.1681/ASN.2005121319 16565257

[47] Palmer BF. Proteinuria as a therapeutic target in patients with chronic kidney disease. Am J Nephrol. (2007) 27:287–93. 10.1159/000101958 17457028

[48] Neumann J Ligtenberg G Klein II Koomans HA Blankestijn PJ. Sympathetic hyperactivity in chronic kidney disease: pathogenesis, clinical relevance, and treatment. Kidney Int. (2004) 65:1568–76. 10.1111/j.1523-1755.2004.00552.x 15086894

[49] Pohl MA Blumenthal S Cordonnier DJ De Alvaro F Deferrari G Eisner G . Independent and additive impact of blood pressure control and angiotensin II receptor blockade on renal outcomes in the irbesartan diabetic nephropathy trial: clinical implications and limitations. J Am Soc Nephrol. (2005) 16:3027–37. 10.1681/ASN.2004110919 16120823

[50] Chen X Wang H Chen L. Effects of astragalus injection on antioxidant capacity and vascular elasticity in patients with renal hypertension. J Hainan Med Coll. (2015) 21:375–7.

[51] Zhu Y. Antagonistic effect of astragaloside IV on renal interstitial fibrosis. Liaoning J Trad Chin Med. (2014) 41:2700–2.

[52] Zhang X. Study on the intervention effect and Mechanism of warming Yang, purging turbidity and dredging collaterals on Renal Interstitial Fibrosis in Rats with chronic Renal failure. Chin Herb Med. (2019) 50:2133–8.

[53] Fu W. Astragaloside IV promotes angiogenesis in rats with myocardial infarction by regulating the PKD1-HDAC5-VEGF pathway. Chin J Pathophysiol. (2018) 34:643–9.

[54] Wang J. Effects of astragaloside IV on myocardial fibrosis and inflammation in hypertensive rats. North Pharm. (2020) 17:187–8.

[55] Wei H. Research overview of Huangqi injection in the treatment of chronic cardiac insufficiency. Chin Pract Med. (2012) 7:171.

