# Supplementary material for: Efficacy of Huangqi Injection in the Treatment of Hypertensive Nephropathy: A Systematic Review and Meta-Analysis
Source: Front Med (Lausanne). 2022 Apr 25;9:838256. doi: 10.3389/fmed.2022.838256 (PMC9081808; doi:10.3389/fmed.2022.838256)
Supplement: Supplementary file 1 [file Data_Sheet_1.PDF]

Supplementary Materials for

“Efficacy and Safety of Huangqi Injection in the treatment of Hypertensive Nephropathy : A Systematic Review and Meta-Analysis”

ZhongChi Xu[1,†], LiChao Qian[2,†], RuGe Niu[1], Ying Yang[1], ChunLing Liu[1*], Xin Lin[1*]

1 *Jiangsu Provincial Hospital of Chinese Medicine, Affiliated Hospital of Nanjing University of Chinese Medicine, Nanjing, Jiangsu 210029, China*

2 *Nanjing Hospital of Chinese Medicine, Affiliated Hospital of Nanjing University of Chinese Medicine, Nanjing, Jiangsu 210001, China*

†Both authors contributed equally to this paper.

*Corresponding author:Xin Lin. *E-mail address*: linxin@njucm.edu.cn

Studies included: **8**
Participants included: **908**

Meta-analysis pooling of **Weighted Mean Differences**
using the **random-effects inverse-variance** model
with **DerSimonian-Laird** estimate of tau²

var1 (var2)	Effect	[95% Conf.	Interval]	% Weight
chen (2015)	**-0.440**	**-0.633**	**-0.247**	**11.51**
guo (2017)	**-0.150**	**-0.292**	**-0.008**	**13.69**
huang (2017)	**-0.300**	**-0.475**	**-0.125**	**12.29**
tang (2006)	**-0.143**	**-0.211**	**-0.074**	**16.58**
wu (2007)	**-0.250**	**-0.504**	**0.004**	**9.16**
yang (2015)	**-0.410**	**-0.724**	**-0.096**	**7.30**
zhao (2015)	**-0.450**	**-0.545**	**-0.355**	**15.67**
zhao (2017)	**-0.280**	**-0.420**	**-0.140**	**13.80**
Overall, DL	**-0.294**	**-0.405**	**-0.183**	**100.00**

Test of overall effect = **0**: **z** = **-5.193** p = **0.000**

Heterogeneity measures, calculated from the data
with Conf. Intervals based on **Gamma (random-effects)** distribution for Q

Measure	Value	df	p-value
Cochran's Q	**33.66**	**7**	**0.000**
		—[95% Conf.	Interval]—
H	**2.193**	**1.000**	**3.546**
I² (%)	**79.2%**	**0.0%**	**92.0%**

H = relative excess in Cochran's Q over its degrees-of-freedom
I² = proportion of total variation in effect estimate due to between-study heterogeneity (based on Q)

Heterogeneity variance estimates

Method	tau²
DL	**0.0181**

Supplementary Fig.1 Meta-analysis result of 24h UTP

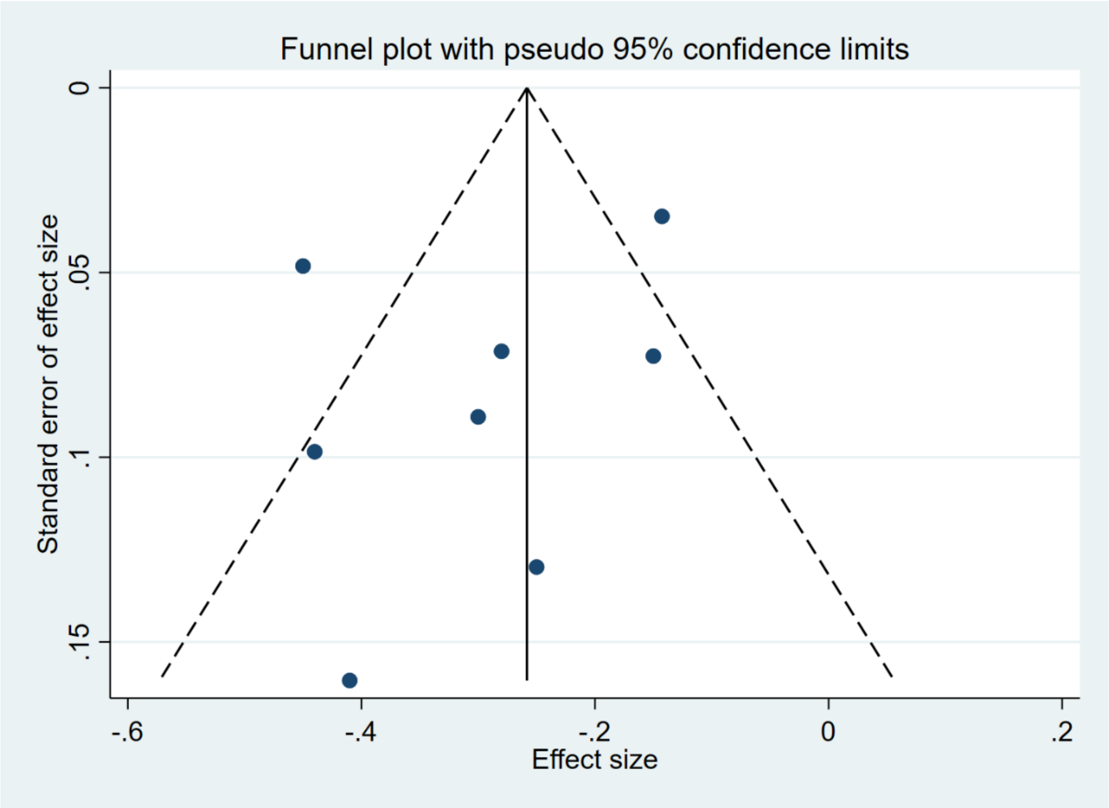

Supplementary Fig.2 Funnel plot for publication bias of 24 h UTP

Egger's test for small-study effects:
Regress standard normal deviate of intervention
effect estimate against its standard error

.
Number of studies = **8** Root MSE = **2.204**

Std_Eff	Coef.	Std. Err.	t	P>|t|	[95% Conf.	Interval]
slope	**-.1610176**	**.1123587**	**-1.43**	**0.202**	**-.4359494**	**.1139142**
bias	**-1.700827**	**1.762445**	**-0.97**	**0.372**	**-6.013375**	**2.611721**

Test of H0: no small-study effects P = **0.372**

Supplementary Fig. 3 Egger's test for 24h UTP

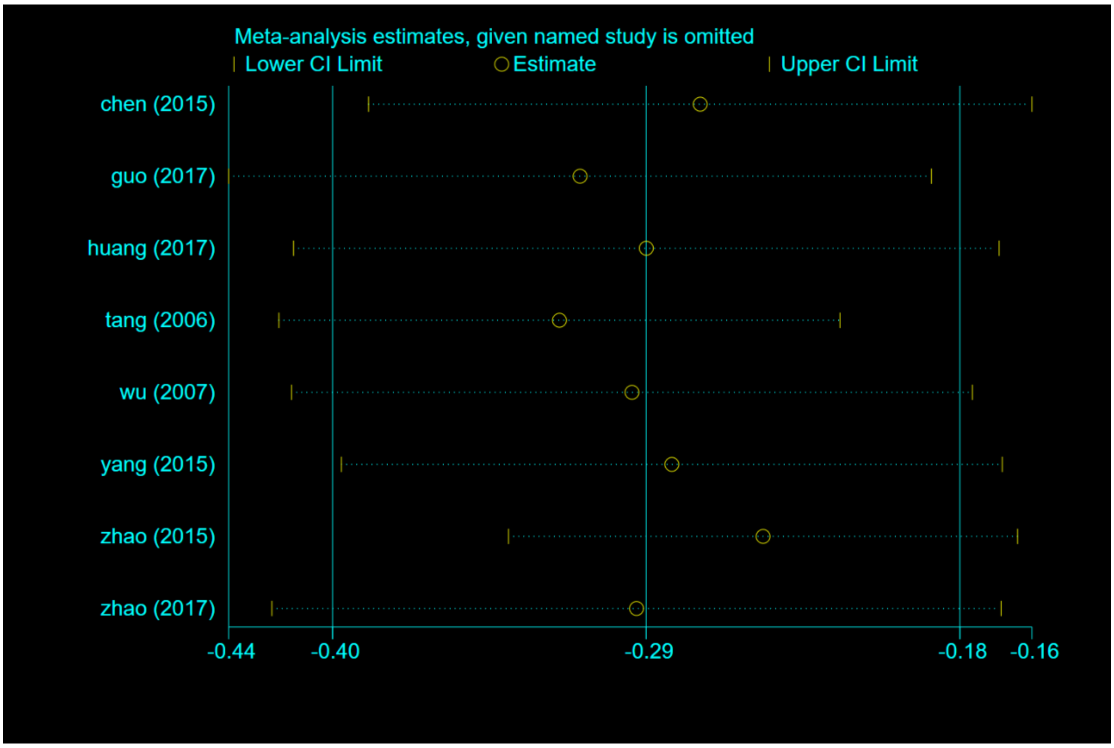

Supplementary Fig. 4 Sensitivity analysis of 24h UTP

```
Meta-analysis regression                               No of studies =    8
                                                       tau^2 method       reml
                                                       tau^2 estimate =      0

Successive values of tau^2 differ by less than 10^-4 :convergence achieved
```

	Coef.	Std. Err.	z	P>|z|	[95% Conf.	Interval]
var9	-.1732052	.1664156	-1.04	0.298	-.4993738	.1529635
var10	-.0100001	.1097002	-0.09	0.927	-.2250084	.2050083
var11	.2501514	.1972454	1.27	0.205	-.1364425	.6367453
var12	(dropped)					
var13	.2132052	.0654306	3.26	0.001	.0849634	.3414469
_cons	-.7201513	.3013447	-2.39	0.017	-1.310776	-.1295265

Supplementary Fig. 5 Regression-analysis result of 24h UTP

Studies included: **8**
Participants included: **908**

Meta-analysis pooling of **Weighted Mean Differences**
using the **random-effects inverse-variance** model
with **DerSimonian-Laird** estimate of tau²

Subgroup and var1 (var2)	Effect	[95% Conf.	Interval]	% Weight
1				
chen (2015)	**-0.440**	**-0.633**	**-0.247**	**11.51**
zhao (2015)	**-0.450**	**-0.545**	**-0.355**	**15.67**
Subgroup, DL	**-0.448**	**-0.533**	**-0.363**	**27.18**
2				
guo (2017)	**-0.150**	**-0.292**	**-0.008**	**13.69**
huang (2017)	**-0.300**	**-0.475**	**-0.125**	**12.29**
tang (2006)	**-0.143**	**-0.211**	**-0.074**	**16.58**
wu (2007)	**-0.250**	**-0.504**	**0.004**	**9.16**
yang (2015)	**-0.410**	**-0.724**	**-0.096**	**7.30**
zhao (2017)	**-0.280**	**-0.420**	**-0.140**	**13.80**
Subgroup, DL	**-0.212**	**-0.287**	**-0.138**	**72.82**
Overall, DL	**-0.294**	**-0.405**	**-0.183**	**100.00**

Tests of subgroup effect size = **0**:

1	**z** = **-10.339**	p = **0.000**
2	**z** = **-5.604**	p = **0.000**
Overall	**z** = **-5.193**	p = **0.000**

Cochran's Q statistics for heterogeneity
(other heterogeneity measures are stored in matrices **r(ovstats)** and **r(bystats)**)

Measure	Value	df	p-value	I²
1	**0.01**	**1**	**0.927**	**0.0%**
2	**7.36**	**5**	**0.195**	**32.1%**
Overall	**33.66**	**7**	**0.000**	**79.2%**
Between	**16.78**	**1**	**0.000**	

Note: between-subgroup heterogeneity calculated using DL subgroup weights

.

Supplementary Fig. 6 Meta-analysis result of subgroups analysis of 24h UTP

Studies included: **4**
Participants included: **442**

Meta-analysis pooling of **Weighted Mean Differences**
using the **random-effects inverse-variance** model
with **DerSimonian-Laird** estimate of tau²

var1 (var2)	Effect	[95% Conf.	Interval]	% Weight
huang (2011)	**-12.340**	**-17.204**	**-7.476**	**31.51**
huang (2017)	**-14.100**	**-18.173**	**-10.027**	**33.51**
ji (2006)	**-27.730**	**-54.923**	**-0.537**	**4.46**
zhao (2017)	**-23.560**	**-28.800**	**-18.320**	**30.52**
Overall, DL	**-17.041**	**-23.141**	**-10.942**	**100.00**

Test of overall effect = **0**: **z** = **-5.476** p = **0.000**

Heterogeneity measures, calculated from the data
with Conf. Intervals based on **Gamma (random-effects)** distribution for Q

Measure	Value	df	p-value
Cochran's Q	**11.71**	**3**	**0.008**
		[95% Conf.	Interval]
H	**1.976**	**1.000**	**3.658**
I² (%)	**74.4%**	**0.0%**	**92.5%**

H = relative excess in Cochran's Q over its degrees-of-freedom
I² = proportion of total variation in effect estimate due to between-study heterogeneity (based on Q)

Heterogeneity variance estimates

Method	tau²
DL	**24.5828**

Supplementary Fig. 7 Meta-analysis result of mALB

Egger's test for small-study effects:
Regress standard normal deviate of intervention
effect estimate against its standard error

.
Number of studies = **4** Root MSE = **2.247**

Std_Eff	Coef.	Std. Err.	t	P>|t|	[95% Conf.	Interval]
slope	**-12.4539**	**7.227721**	**-1.72**	**0.227**	**-43.55227**	**18.64448**
bias	**-1.500431**	**2.653386**	**-0.57**	**0.629**	**-12.91703**	**9.916167**

Test of H0: no small-study effects P = **0.629**

.

Supplementary Fig. 8 Egger's test for mALB

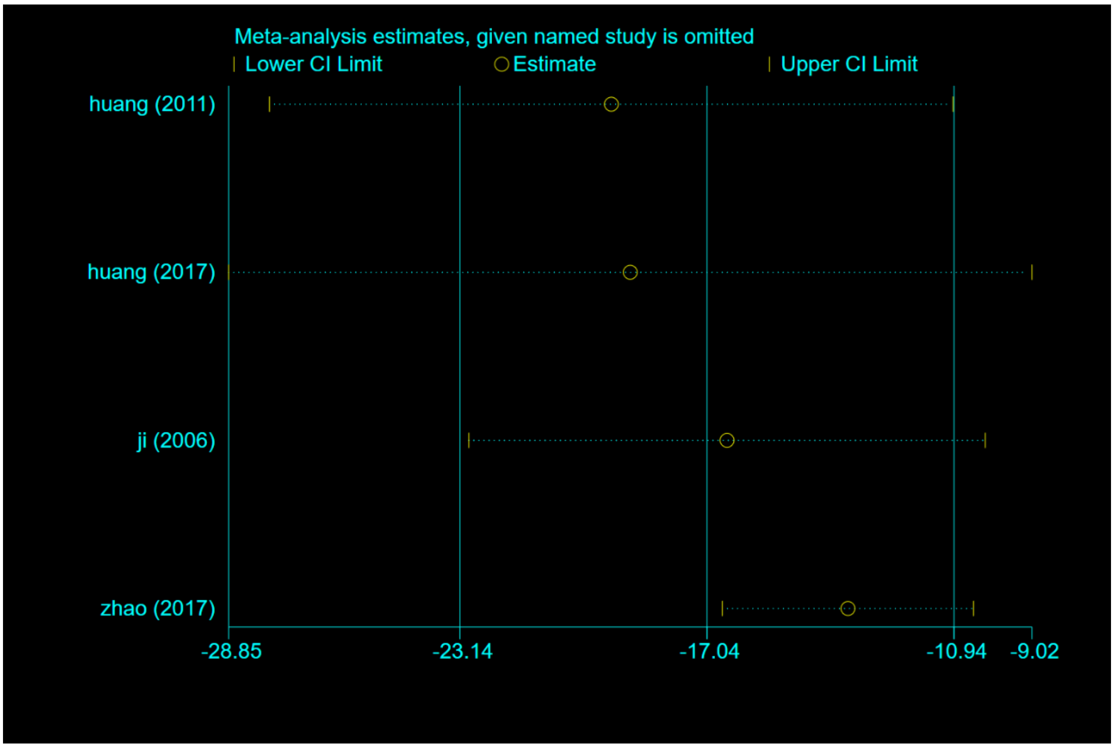

Supplementary Fig. 9 Sensitivity analysis of mALB

Studies included: **4**
Participants included: **442**

Meta-analysis pooling of **Weighted Mean Differences**
using the **random-effects inverse-variance** model
with **DerSimonian-Laird** estimate of tau²

Subgroup and var1 (var2)	Effect	[95% Conf.	Interval]	% Weight
1				
huang (2011)	-12.340	-17.204	-7.476	31.51
huang (2017)	-14.100	-18.173	-10.027	33.51
Subgroup, DL	-13.375	-16.497	-10.252	65.02
2				
ji (2006)	-27.730	-54.923	-0.537	4.46
zhao (2017)	-23.560	-28.800	-18.320	30.52
Subgroup, DL	-23.709	-28.854	-18.564	34.98
Overall, DL	-17.041	-23.141	-10.942	100.00

Tests of subgroup effect size = **0**:

1	z = -8.395	p = 0.000
2	z = -9.032	p = 0.000
Overall	z = -5.476	p = 0.000

Cochran's Q statistics for heterogeneity
(other heterogeneity measures are stored in matrices **r(ovstats)** and **r(bystats)**)

Measure	Value	df	p-value	I²
1	0.30	1	0.587	0.0%
2	0.09	1	0.768	0.0%
Overall	11.71	3	0.008	74.4%
Between	11.33	1	0.001	

Note: between-subgroup heterogeneity calculated using DL subgroup weights

.

Supplementary Fig. 10 Meta-analysis result of subgroups analysis of mALB

Studies included: **5**
Participants included: **531**

Meta-analysis pooling of **Weighted Mean Differences**
using the **random-effects inverse-variance** model
with **DerSimonian-Laird** estimate of tau²

var1 (var2)	Effect	[95% Conf.	Interval]	% Weight
guo (2017)	-61.400	-69.848	-52.952	22.23
zhao (2015)	-61.300	-69.095	-53.505	22.29
chen (2015)	-8.790	-14.299	-3.281	22.46
dong (2002)	-29.100	-56.646	-1.554	19.01
he (2004)	-39.770	-89.240	9.700	14.01
Overall, DL	-40.392	-70.391	-10.393	100.00

Test of overall effect = **0**: **z** = **-2.639** p = **0.008**

Heterogeneity measures, calculated from the data
with Conf. Intervals based on **Gamma (random-effects)** distribution for Q

Measure	Value	df	p-value
Cochran's Q	168.14	4	0.000
		—[95% Conf.	Interval]—
H	6.484	1.164	12.243
I^2 (%)	97.6%	26.1%	99.3%

H = relative excess in Cochran's Q over its degrees-of-freedom
I^2 = proportion of total variation in effect estimate due to between-study heterogeneity (based on Q)

Heterogeneity variance estimates

Method	tau²
DL	1.0e+03

Supplementary Fig. 11 Meta-analysis result of Scr

Egger's test for small-study effects:
Regress standard normal deviate of intervention
effect estimate against its standard error

.
Number of studies = **5** Root MSE = **7.206**

Std_Eff	Coef.	Std. Err.	t	P>|t|	[95% Conf.	Interval]
slope	-22.66058	27.28169	-0.83	0.467	-109.4831	64.16195
bias	-2.980866	6.103437	-0.49	0.659	-22.40473	16.44299

Test of H0: no small-study effects P = **0.659**

Supplementary Fig. 12 Egger's test for Scr

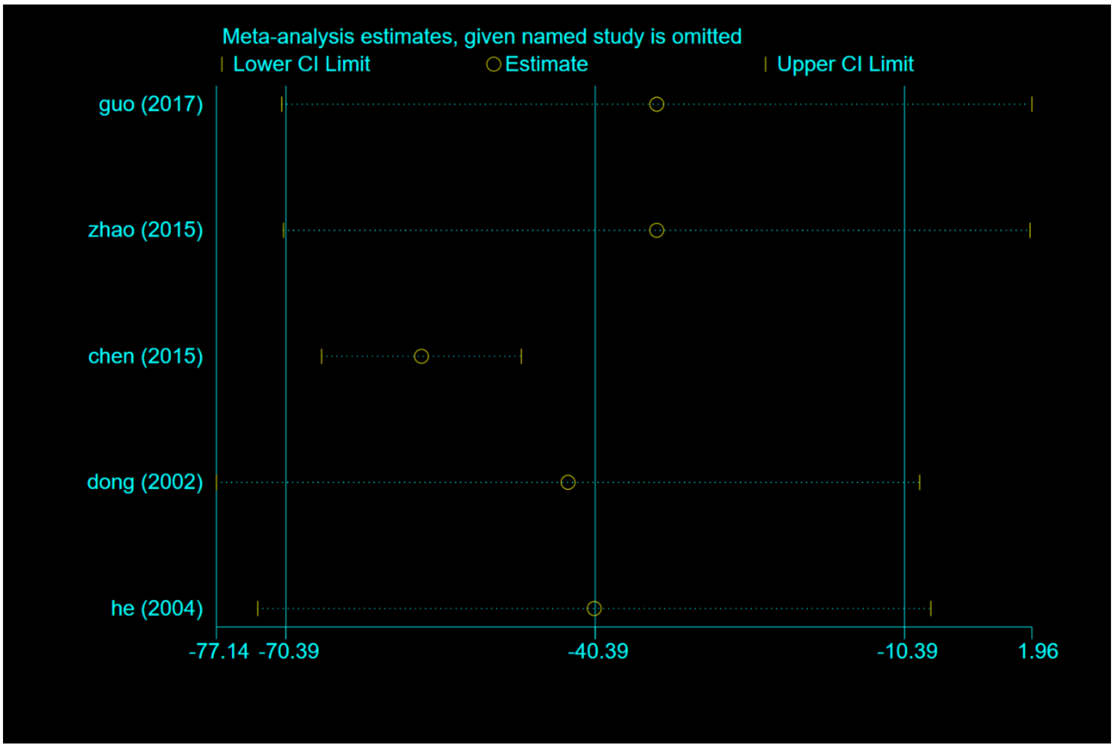

Supplementary Fig. 13 Sensitivity analysis of Scr

Studies included: **4**
Participants included: **442**

Meta-analysis pooling of **Weighted Mean Differences**
using the **random-effects inverse-variance** model
with **DerSimonian-Laird** estimate of tau²

Subgroup and var1 (var2)	Effect	[95% Conf.	Interval]	% Weight
1				
huang (2011)	**-12.340**	**-17.204**	**-7.476**	**31.51**
huang (2017)	**-14.100**	**-18.173**	**-10.027**	**33.51**
Subgroup, DL	**-13.375**	**-16.497**	**-10.252**	**65.02**
2				
ji (2006)	**-27.730**	**-54.923**	**-0.537**	**4.46**
zhao (2017)	**-23.560**	**-28.800**	**-18.320**	**30.52**
Subgroup, DL	**-23.709**	**-28.854**	**-18.564**	**34.98**
Overall, DL	**-17.041**	**-23.141**	**-10.942**	**100.00**

Tests of subgroup effect size = **0**:

1	**z** = **-8.395**	p = **0.000**
2	**z** = **-9.032**	p = **0.000**
Overall	**z** = **-5.476**	p = **0.000**

Cochran's Q statistics for heterogeneity
(other heterogeneity measures are stored in matrices **r(ovstats)** and **r(bystats)**)

Measure	Value	df	p-value	I²
1	**0.30**	**1**	**0.587**	**0.0%**
2	**0.09**	**1**	**0.768**	**0.0%**
Overall	**11.71**	**3**	**0.008**	**74.4%**
Between	**11.33**	**1**	**0.001**	

Note: between-subgroup heterogeneity calculated using DL subgroup weights

.

Supplementary Fig. 14 Meta-analysis result of subgroups analysis of Scr

Studies included: **7**
Participants included: **810**

Meta-analysis pooling of **Weighted Mean Differences**
using the **random-effects inverse-variance** model
with **DerSimonian-Laird** estimate of tau²

var1 (var2)	Effect	[95% Conf.	Interval]	% Weight
chen (2015)	**-11.900**	**-17.343**	**-6.457**	**13.33**
guo (2017)	**-18.890**	**-20.046**	**-17.734**	**15.55**
han (2011)	**-4.500**	**-10.878**	**1.878**	**12.62**
song (2019)	**-9.580**	**-10.948**	**-8.212**	**15.50**
yang (2015)	**-4.950**	**-9.244**	**-0.656**	**14.12**
zhao (2017)	**-13.600**	**-17.134**	**-10.066**	**14.59**
zhaoYJ (2015)	**-1.720**	**-5.739**	**2.299**	**14.30**
Overall, DL	**-9.504**	**-14.642**	**-4.366**	**100.00**

Test of overall effect = **0**: **z** = **-3.625** p = **0.000**

Heterogeneity measures, calculated from the data
with Conf. Intervals based on **Gamma (random-effects)** distribution for Q

Measure	Value	df	p-value
Cochran's Q	**170.72**	**6**	**0.000**
		[95% Conf.	Interval]
H	**5.334**	**1.060**	**9.919**
I² (%)	**96.5%**	**11.0%**	**99.0%**

H = relative excess in Cochran's Q over its degrees-of-freedom
I² = proportion of total variation in effect estimate due to between-study heterogeneity (based on Q)

Heterogeneity variance estimates

Method	tau²
DL	**43.8563**

Supplementary Fig. 15 Meta-analysis result of SBP

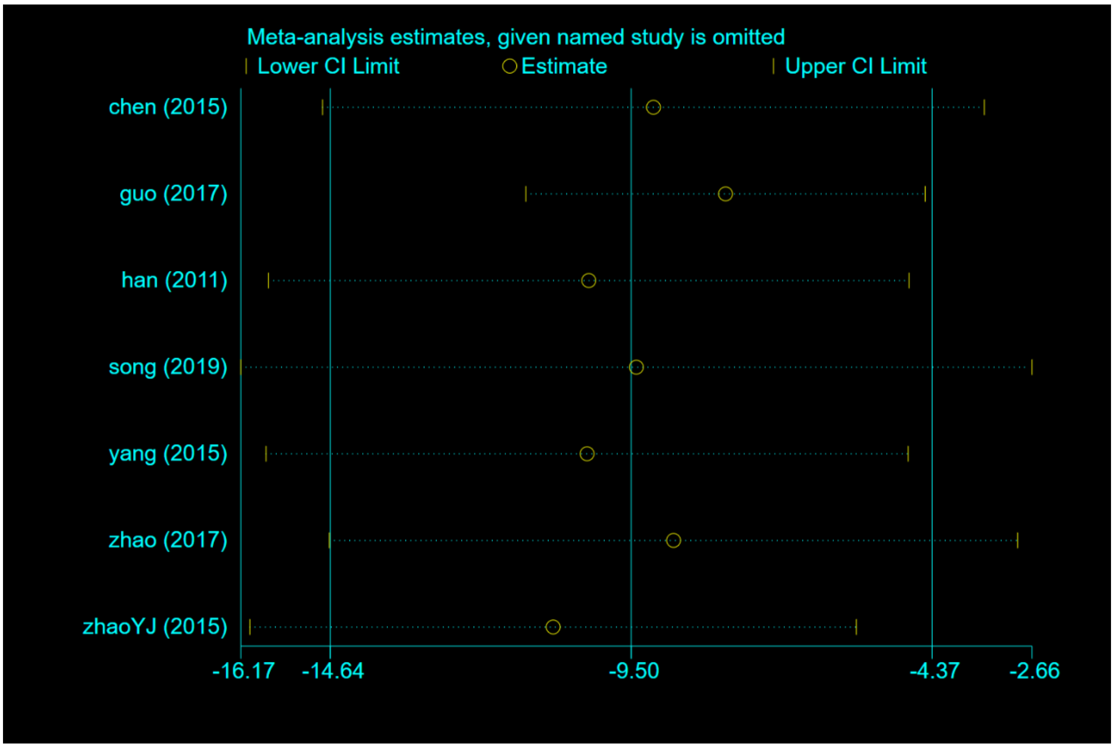

Supplementary Fig. 16 Sensitivity analysis of SBP

Egger's test for small-study effects:
Regress standard normal deviate of intervention
  effect estimate against its standard error

.
Number of studies = 7   Root MSE = **4.826**

Std_Eff	Coef.	Std. Err.	t	P>|t|	[95% Conf.	Interval]
slope	**-18.20857**	**3.500145**	**-5.20**	**0.003**	**-27.20598**	**-9.211162**
bias	**4.906297**	**3.215138**	**1.53**	**0.188**	**-3.358477**	**13.17107**

Test of H0: no small-study effects   P = **0.188**

Supplementary Fig. 17 Egger’s test for SBP

Studies included: **7**
Participants included: **810**

Meta-analysis pooling of **Weighted Mean Differences**
using the **random-effects inverse-variance** model
with **DerSimonian-Laird** estimate of tau²

Subgroup and var1 (var2)	Effect	[95% Conf.	Interval]	% Weight
1				
chen (2015)	-11.900	-17.343	-6.457	13.33
guo (2017)	-18.890	-20.046	-17.734	15.55
song (2019)	-9.580	-10.948	-8.212	15.50
zhao (2017)	-13.600	-17.134	-10.066	14.59
Subgroup, DL	-13.570	-19.506	-7.633	58.96
2				
han (2011)	-4.500	-10.878	1.878	12.62
yang (2015)	-4.950	-9.244	-0.656	14.12
zhaoYJ (2015)	-1.720	-5.739	2.299	14.30
Subgroup, DL	-3.451	-6.116	-0.785	41.04
Overall, DL	-9.504	-14.642	-4.366	100.00

Tests of subgroup effect size = **0**:

1	z = -4.480	p = 0.000
2	z = -2.537	p = 0.011
Overall	z = -3.625	p = 0.000

Cochran's Q statistics for heterogeneity
(other heterogeneity measures are stored in matrices **r(ovstats)** and **r(bystats)**)

Measure	Value	df	p-value	I²
1	105.57	3	0.000	97.2%
2	1.28	2	0.526	0.0%
Overall	170.72	6	0.000	96.5%
Between	9.29	1	0.002	

Note: between-subgroup heterogeneity calculated using DL subgroup weights

.

Supplementary Fig. 18 Meta-analysis result of subgroups analysis of SBP

Studies included: **7**
Participants included: **810**

Meta-analysis pooling of **Weighted Mean Differences**
using the **random-effects inverse-variance** model
with **DerSimonian-Laird** estimate of tau²

var1 (var2)	Effect	[95% Conf.	Interval]	% Weight
chen (2015)	**-4.830**	**-8.131**	**-1.529**	**12.01**
guo (2017)	**-7.310**	**-9.707**	**-4.913**	**17.03**
han (2011)	**-3.750**	**-7.532**	**0.032**	**10.05**
song (2019)	**-5.830**	**-8.060**	**-3.600**	**18.17**
yang (2015)	**-2.770**	**-4.573**	**-0.967**	**21.36**
zhao (2017)	**-4.040**	**-6.563**	**-1.517**	**16.21**
zhaoYJ (2015)	**-1.560**	**-7.388**	**4.268**	**5.17**
Overall, DL	**-4.588**	**-6.036**	**-3.140**	**100.00**

Test of overall effect = **0**: **z** = **-6.210** p = **0.000**

Heterogeneity measures, calculated from the data
with Conf. Intervals based on **Gamma (random-effects)** distribution for Q

Measure	Value	df	p-value
Cochran's Q	**11.47**	**6**	**0.075**
		[95% Conf.	Interval]
H	**1.383**	**1.000**	**2.171**
I² (%)	**47.7%**	**0.0%**	**78.8%**

H = relative excess in Cochran's Q over its degrees-of-freedom
I² = proportion of total variation in effect estimate due to between-study heterogeneity (based on Q)

Heterogeneity variance estimates

Method	tau²
DL	**1.7097**

Supplementary Fig. 19 Meta-analysis result of DBP

Egger's test for small-study effects:
Regress standard normal deviate of intervention
effect estimate against its standard error

.
Number of studies = **7** Root MSE = **1.512**

Std_Eff	Coef.	Std. Err.	t	P>|t|	[95% Conf.	Interval]
slope	**-4.852767**	**2.464015**	**-1.97**	**0.106**	**-11.18672**	**1.481186**
bias	**.2456837**	**1.853388**	**0.13**	**0.900**	**-4.518601**	**5.009968**

Test of H0: no small-study effects P = **0.900**

Supplementary Fig. 20 Egger's test for DBP

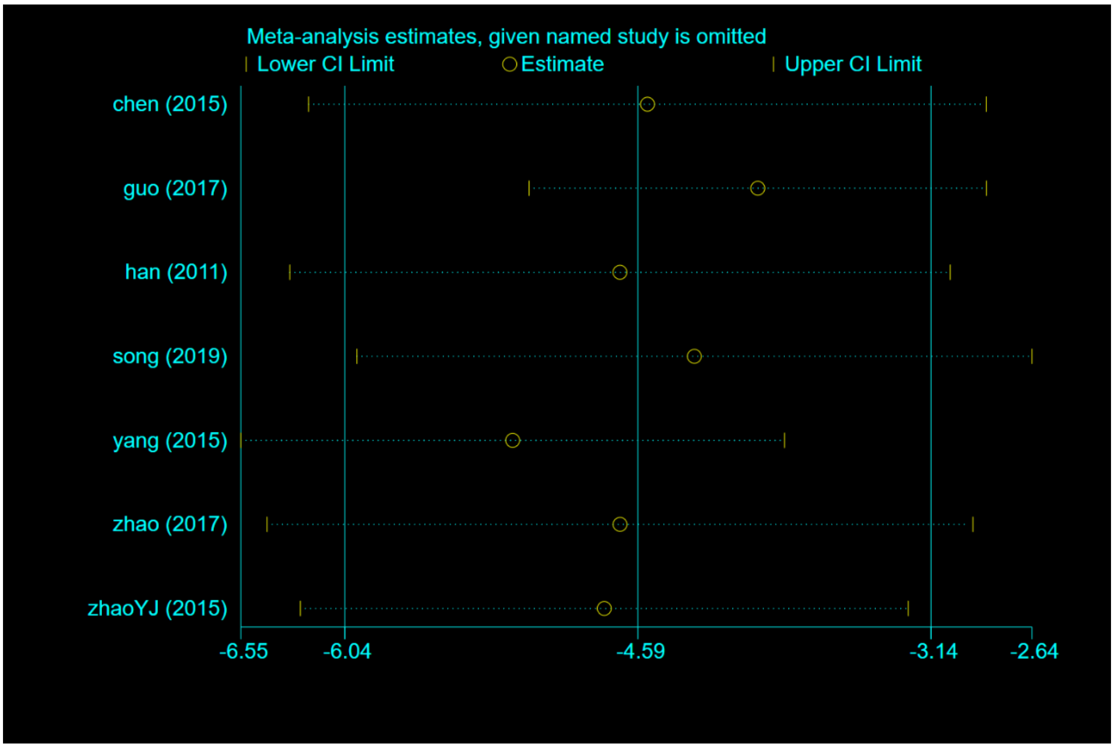

Supplementary Fig. 21 Sensitivity analysis of DBP

Studies included: **7**
Participants included: **810**

Meta-analysis pooling of **Weighted Mean Differences**
using the **random-effects inverse-variance** model
with **DerSimonian-Laird** estimate of tau²

Subgroup and var1 (var2)	Effect	[95% Conf.	Interval]	% Weight
1				
chen (2015)	-4.830	-8.131	-1.529	12.01
guo (2017)	-7.310	-9.707	-4.913	17.03
song (2019)	-5.830	-8.060	-3.600	18.17
zhao (2017)	-4.040	-6.563	-1.517	16.21
Subgroup, DL	-5.623	-7.033	-4.213	63.42
2				
han (2011)	-3.750	-7.532	0.032	10.05
yang (2015)	-2.770	-4.573	-0.967	21.36
zhaoYJ (2015)	-1.560	-7.388	4.268	5.17
Subgroup, DL	-2.851	-4.418	-1.283	36.58
Overall, DL	-4.588	-6.036	-3.140	100.00

Tests of subgroup effect size = **0**:

1	z = -7.815	p = 0.000
2	z = -3.565	p = 0.000
Overall	z = -6.210	p = 0.000

Cochran's Q statistics for heterogeneity
(other heterogeneity measures are stored in matrices **r(ovstats)** and **r(bystats)**)

Measure	Value	df	p-value	I²
1	3.67	3	0.300	18.2%
2	0.41	2	0.813	0.0%
Overall	11.47	6	0.075	47.7%
Between	6.64	1	0.010	

Note: between-subgroup heterogeneity calculated using DL subgroup weights

.

Supplementary Fig. 22 Meta-analysis result of subgroups analysis of DBP

```
Studies included: 4
Participants included: 422

Meta-analysis pooling of  Weighted Mean Differences
using the random-effects inverse-variance model
with DerSimonian-Laird estimate of tau²
```

var1 (var2)	Effect	[95% Conf.	Interval]	% Weight
guo (2017)	-0.860	-1.076	-0.644	25.61
huang (2011)	-0.450	-0.626	-0.274	27.71
huang (2017)	-0.900	-1.216	-0.584	20.28
zhao (2015)	-0.830	-1.031	-0.629	26.40
Overall, DL	-0.747	-0.975	-0.518	100.00

```
Test of overall effect = 0:  z =  -6.406   p = 0.000

Heterogeneity measures, calculated from the data
with Conf. Intervals based on Gamma (random-effects) distribution for Q
```

Measure	Value	df	p-value
Cochran's Q	12.98	3	0.005
		—[95% Conf.	Interval]—
H	2.080	1.000	3.720
I² (%)	76.9%	0.0%	92.8%

```
H = relative excess in Cochran's Q over its degrees-of-freedom
I² = proportion of total variation in effect estimate due to between-study heterogeneity (based on Q)

Heterogeneity variance estimates
```

Method	tau²
DL	0.0409

.

Supplementary Fig. 23 Meta-analysis result of Cys-c

```
Egger's test for small-study effects:
Regress standard normal deviate of intervention
  effect estimate against its standard error

.
Number of studies =   4                          Root MSE      =    1.904
```

Std_Eff	Coef.	Std. Err.	t	P>|t|	[95% Conf.	Interval]
slope	-.0542323	.5293197	-0.10	0.928	-2.331711	2.223246
bias	-6.133252	4.87931	-1.26	0.336	-27.12723	14.86072

```
Test of H0: no small-study effects          P = 0.336
```

Supplementary Fig.24 Egger's test for Cys-c

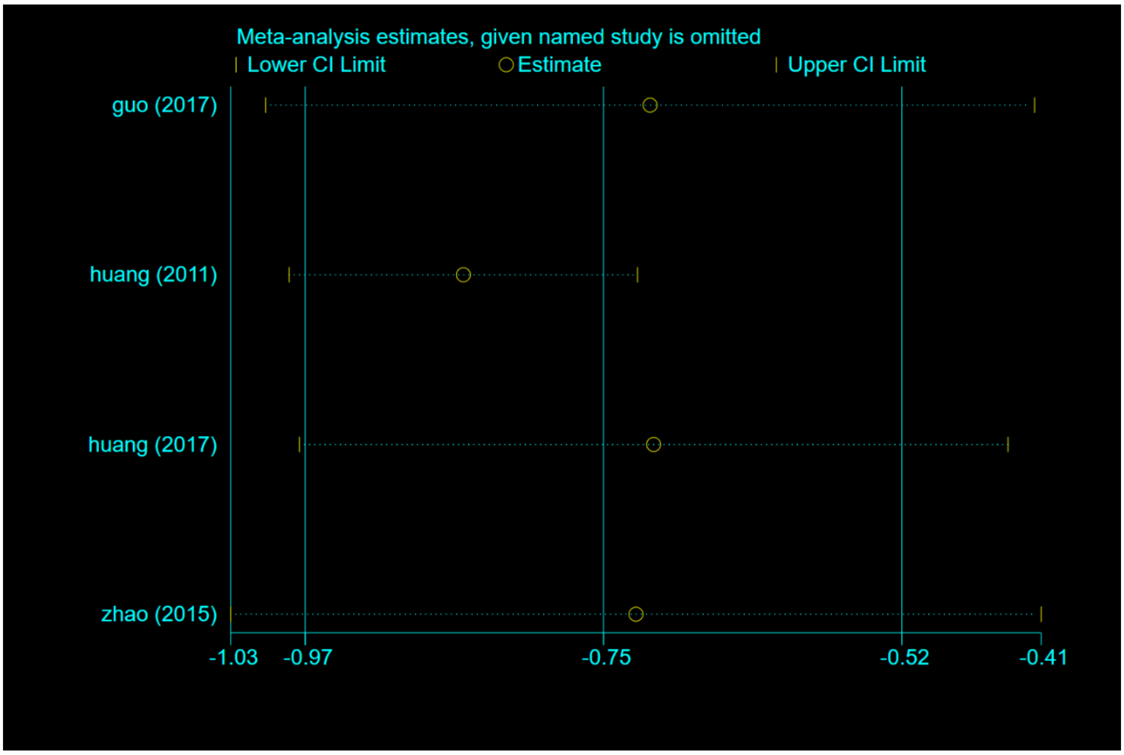

Supplementary Fig. 25 Sensitivity analysis of Cys-c

Studies included: **3**
Participants included: **332**

Meta-analysis pooling of **Weighted Mean Differences**
using the **random-effects inverse-variance** model
with **DerSimonian-Laird** estimate of tau²

var1 (var2)	Effect	[95% Conf.	Interval]	% Weight
guo (2017)	**-0.860**	**-1.076**	**-0.644**	**38.21**
huang (2017)	**-0.900**	**-1.216**	**-0.584**	**17.78**
zhao (2015)	**-0.830**	**-1.031**	**-0.629**	**44.01**
Overall, DL	**-0.854**	**-0.987**	**-0.721**	**100.00**

Test of overall effect = **0**: **z = -12.553** p = **0.000**

Heterogeneity measures, calculated from the data
with Conf. Intervals based on **Gamma (random-effects)** distribution for Q

Measure	Value	df	p-value
Cochran's Q	**0.14**	**2**	**0.933**
		—[95% Conf.	Interval]—
H	**0.264**	**1.000**	**0.881**
I² (%)	**0.0%**	**0.0%**	**0.0%**

H = relative excess in Cochran's Q over its degrees-of-freedom
I² = proportion of total variation in effect estimate due to between-study heterogeneity (based on Q)

Heterogeneity variance estimates

Method	tau²
DL	**0.0000**

.

Supplementary Fig. 26 Meta-analysis result of Cys-c after exclude

```
Studies included: 5
Participants included: 483

Meta-analysis pooling of  Weighted Mean Differences
using the random-effects inverse-variance model
with DerSimonian-Laird estimate of tau²
```

var1 (var2)	Effect	[95% Conf.	Interval]	% Weight
dong (2002)	-2.140	-3.588	-0.692	21.56
guo (2017)	-5.400	-6.909	-3.891	21.35
he (2004)	-8.600	-12.639	-4.561	12.19
zhao (2015)	-5.100	-6.447	-3.753	21.89
zhao (2017)	-1.630	-2.600	-0.660	23.00
Overall, DL	-4.155	-6.152	-2.157	100.00

```
Test of overall effect = 0:  z =  -4.077  p = 0.000

Heterogeneity measures, calculated from the data
with Conf. Intervals based on Gamma (random-effects) distribution for Q
```

Measure	Value	df	p-value
Cochran's Q	34.75	4	0.000
		—[95% Conf.	Interval]—
H	2.948	1.000	5.142
I² (%)	88.5%	0.0%	96.2%

```
H = relative excess in Cochran's Q over its degrees-of-freedom
I² = proportion of total variation in effect estimate due to between-study heterogeneity (based on Q)

Heterogeneity variance estimates
```

Method	tau²
DL	4.2712

Supplementary Fig. 27 Meta-analysis result of BUN

```
Egger's test for small-study effects:
Regress standard normal deviate of intervention
  effect estimate against its standard error

.
Number of studies =  5                              Root MSE      =    2.44
```

Std_Eff	Coef.	Std. Err.	t	P>|t|	[95% Conf.	Interval]
slope	.2412832	2.220401	0.11	0.920	-6.825022	7.307589
bias	-5.27867	3.132881	-1.68	0.191	-15.2489	4.691555

```
Test of H0: no small-study effects          P = 0.191
```

Supplementary Fig. 28 Egger’s test for BUN

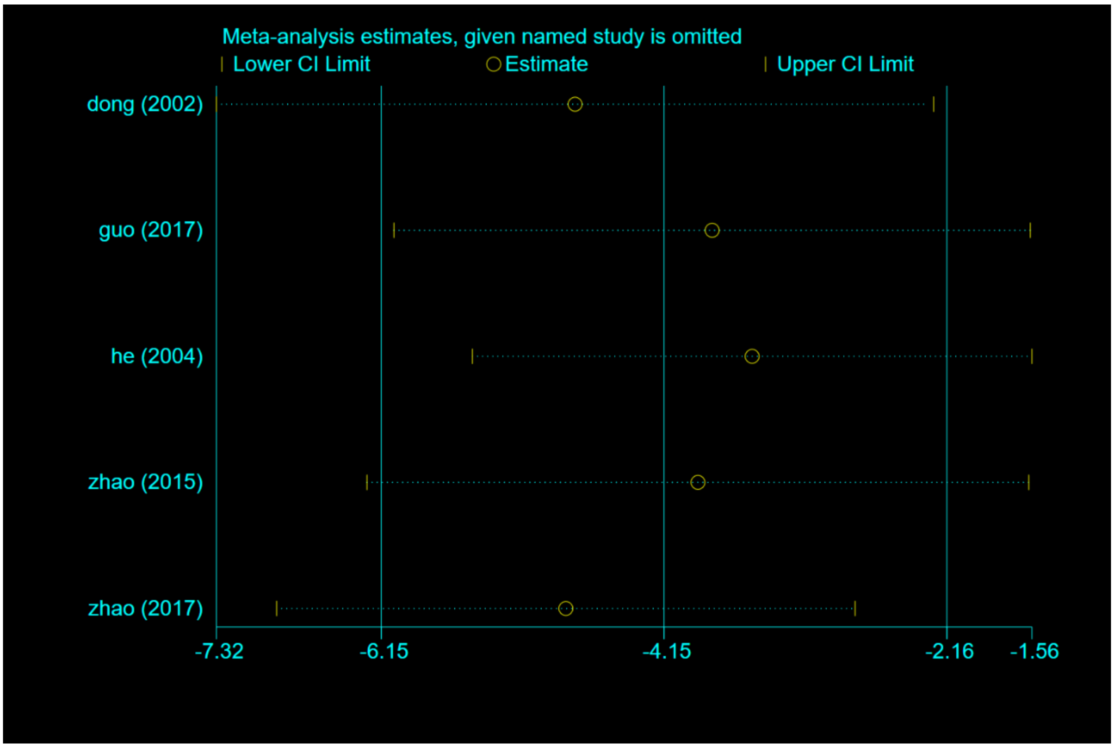

Supplementary Fig. 29 Sensitivity analysis of BUN
