# Supplementary material for: Efficacy of Huangqi Injection in the Treatment of Hypertensive Nephropathy: A Systematic Review and Meta-Analysis
Source: Front Med (Lausanne). 2022 Apr 25;9:838256. doi: 10.3389/fmed.2022.838256 (PMC9081808; doi:10.3389/fmed.2022.838256)
Supplement: Supplementary file 2 [file Data_Sheet_2.PDF]

Efficacy and Safety of Huangqi Injection in the treatment of Hypertensive Nephropathy : A Systematic Review and Meta-Analysis

Study	Source	Species，concentration	Quality control reported? (Y/N)	Chemical analysis reported? (Y/N)
Chen.(2015)	Not mentioned	Not mentioned	N	N
Dong.(2002)	Not mentioned	Not mentioned	N	N
Guo.(2017)	Shenwei Pharmaceutical Co., Ltd.	Astragalus mongholicus Bunge,60g	Y-Prepared according to The Seventeenth Book of the Pharmaceutical Standards of the Ministry of Health of the People's Republic of China for Prescriptions of Traditional Chinese Medicine WS3-B-3335-98	Y-HPLC
Han.(2011)	Chengdu Diao Jiuhong Pharmaceutical Co., Ltd.	Astragalus mongholicus Bunge,80g	Y-Prepared according to The Seventeenth Book of the Pharmaceutical Standards of the Ministry of Health of the People's Republic of China for Prescriptions of Traditional Chinese Medicine WS3-B-3335-98	Y-HPLC
He.(2004)	Not mentioned	Not mentioned	N	N
Huang.(2011)	Chengdu Diao Jiuhong Pharmaceutical Co., Ltd.	Astragalus mongholicus Bunge,60g	Y-Prepared according to The Seventeenth Book of the Pharmaceutical Standards of the Ministry of Health of the People's Republic of China for Prescriptions of	Y-HPLC

			Traditional Chinese Medicine WS3-B-3335-98	
Huang.(2017)	Heilongjiang Zhenbaodao Pharmaceutical Co., Ltd.	Astragalus mongholicus Bunge,60g	Y-Prepared according to The Seventeenth Book of the Pharmaceutical Standards of the Ministry of Health of the People's Republic of China for Prescriptions of Traditional Chinese Medicine WS3-B-3335-98	Y-HPLC
Ji.(2006)	Chengdu Diao Jiuhong Pharmaceutical Co., Ltd.	Astragalus mongholicus Bunge,50g	Y-Prepared according to The Seventeenth Book of the Pharmaceutical Standards of the Ministry of Health of the People's Republic of China for Prescriptions of Traditional Chinese Medicine WS3-B-3335-98	Y-HPLC
Song.(2019)	Shenwei Pharmaceutical Co., Ltd.	Astragalus mongholicus Bunge,60g	Y-Prepared according to The Seventeenth Book of the Pharmaceutical Standards of the Ministry of Health of the People's Republic of China for Prescriptions of Traditional Chinese Medicine WS3-B-3335-98	Y-HPLC
Tang.(2006)	Chengdu Diao Jiuhong Pharmaceutical Co., Ltd.	Astragalus mongholicus Bunge,120g	Y-Prepared according to The Seventeenth Book of the Pharmaceutical Standards of the	Y-HPLC

			Ministry of Health of the People's Republic of China for Prescriptions of Traditional Chinese Medicine WS3-B-3335-98	
Wu.(2007)	Not mentioned	Not mentioned	N	N
Yang.(2015)	Chengdu Diao Jiuhong Pharmaceutical Co., Ltd.	Astragalus mongholicus Bunge,80g	Y-Prepared according to The Seventeenth Book of the Pharmaceutical Standards of the Ministry of Health of the People's Republic of China for Prescriptions of Traditional Chinese Medicine WS3-B-3335-98	Y-HPLC
Zhao.(2015)	Zhengda Qingchunbao Pharmaceutical Co., Ltd.	Astragalus mongholicus Bunge,60g	Y-Prepared according to The Seventeenth Book of the Pharmaceutical Standards of the Ministry of Health of the People's Republic of China for Prescriptions of Traditional Chinese Medicine WS3-B-3335-98	Y-HPLC
Zhao.(2017)	Shenwei Pharmaceutical Co., Ltd.	Astragalus mongholicus Bunge,40g	Y-Prepared according to The Seventeenth Book of the Pharmaceutical Standards of the Ministry of Health of the People's Republic of China for Prescriptions of Traditional Chinese Medicine WS3-B-3335-98	Y-HPLC

ZhaoYJ.(2015)	Chengdu Diao Jiuhong Pharmaceutical Co., Ltd.	Astragalus mongholicus Bunge,80g	Y-Prepared according to The Seventeenth Book of the Pharmaceutical Standards of the Ministry of Health of the People's Republic of China for Prescriptions of Traditional Chinese Medicine WS3-B-3335-98	Y-HPLC
