# Supplementary material for: Efficacy of Huangqi Injection in the Treatment of Hypertensive Nephropathy: A Systematic Review and Meta-Analysis
Source: Front Med (Lausanne). 2022 Apr 25;9:838256. doi: 10.3389/fmed.2022.838256 (PMC9081808; doi:10.3389/fmed.2022.838256)
Supplement: Supplementary file 3 [file Data_Sheet_3.PDF]

Search Strategy

Database	Search terms	Results
PubMed	((((((((huangqi[Title/Abstract]) OR huangqi injection[Title/Abstract]) OR astragalus[Title/Abstract]))) OR astragalus Injection[Title/Abstract])) AND((hypertension[Mesh]) OR ((((((hypertensive nephropathy[Title/Abstract]) OR hypertension nephropathy[Title/Abstract]) OR hypertensive renal injury[Title/Abstract]) OR hypertensive kidney injury[Title/Abstract]) OR hypertensive renal damage[Title/Abstract]) OR hypertensive kidney damage[Title/Abstract])))	5
Cochrane Library	#1 (hypertension)OR (hypertensive nephropathy) OR (hypertension nephropathy) OR (hypertensive renal injury) OR (hypertensive kidney injury) OR(hypertensive renal damage) OR (hypertensive kidney damage)<br>#2 (huangqi) OR (huangqi injection) OR (astragalus injection) OR (astragalus)<br>#1and#2	22
EMBASE	#1 huangqi:ab,ti OR 'huangqi injection':ab,ti OR astragalus:ab,ti OR 'astragalus injection':ab,ti<br>#2 hypertension:ab,ti OR 'hypertensive nephropathy':ab,ti OR 'hypertension nephropathy':ab,ti OR 'hypertensive renal injury':ab,ti OR 'hypertensive kidney injury':ab,ti OR 'hypertensive renal damage':ab,ti OR 'hypertensive kidney damage':ab,ti<br>#3 #1 and #2	16
CNKI	FT=('高血压肾损害'+'高血压肾病'+'高血压肾功能不全'+'高血压性肾损害')*(‘黄芪注射液’)	873
WanfangData	全部: (("高血压肾损害" + "高血压肾病"+"高血压肾功能不全"+"高血压性肾病") * ("黄芪注射液"))	25
VIP	(U=高血压肾损害 OR 高血压肾病 OR 高血压肾功能不全 OR 高血压性肾病）AND (U=黄芪注射液)	27
CBM	(("黄芪注射液"[常用字段]) OR "黄芪"[常用字段]) AND (((("高血压肾病"[常用字段]) OR "高血压肾损害"[常用字段]) OR "高血压肾功能不全"[常用字段]) OR "高血压性肾病"[常用字段])	34
